# Tumor-specific interendothelial adhesion mediated by FLRT2 facilitates cancer aggressiveness

**DOI:** 10.1172/JCI153626

**Published:** 2022-03-15

**Authors:** Tomofumi Ando, Ikue Tai-Nagara, Yuki Sugiura, Dai Kusumoto, Koji Okabayashi, Yasuaki Kido, Kohji Sato, Hideyuki Saya, Sutip Navankasattusas, Dean Y. Li, Makoto Suematsu, Yuko Kitagawa, Elena Seiradake, Satoru Yamagishi, Yoshiaki Kubota

**Affiliations:** 1 Department of Anatomy,; 2 Department of Surgery,; 3 Department of Biochemistry, and; 4 Department of Cardiology, Keio University School of Medicine, Tokyo, Japan.; 5 Department of Organ & Tissue Anatomy, Hamamatsu University School of Medicine, Hamamatsu, Japan.; 6 Division of Gene Regulation, Institute for Advanced Medical Research, Keio University School of Medicine, Tokyo, Japan.; 7 Department of Medicine, Program in Molecular Medicine,; 8 Department of Oncological Sciences,; 9 Department of Human Genetics,; 10 ARUP Laboratories,; 11 Division of Cardiovascular Medicine, Department of Medicine, and; 12 Department of Cardiology, VA Salt Lake City Health Care System, University of Utah, Salt Lake City, Utah, USA.; 13 Key Laboratory for Human Disease Gene Study, Sichuan Academy of Medical Sciences and Sichuan Provincial People’s Hospital, Chengdu, China.; 14 Department of Biochemistry, University of Oxford, Oxford, United Kingdom.

**Keywords:** Angiogenesis, Vascular Biology, Cancer, Endothelial cells

## Abstract

Blood vessel abnormalization alters cancer cell metabolism and promotes cancer dissemination and metastasis. However, the biological features of the abnormalized blood vessels that facilitate cancer progression and whether they can be targeted therapeutically have not been fully investigated. Here, we found that an axon guidance molecule, fibronectin leucine-rich transmembrane protein 2 (FLRT2), is expressed preferentially in abnormalized vessels of advanced colorectal cancers in humans and that its expression correlates negatively with long-term survival. Endothelial cell–specific deletion of *Flrt2* in mice selectively pruned abnormalized vessels, resulting in a unique metabolic state termed “oxygen-glucose uncoupling,” which suppressed tumor metastasis. Moreover, Flrt2 deletion caused an increase in the number of mature vessels, resulting in a significant increase in the antitumor effects of immune checkpoint blockers. Mechanistically, we found that FLRT2 forms noncanonical interendothelial adhesions that safeguard against oxidative stress through homophilic binding. Together, our results demonstrated the existence of tumor-specific interendothelial adhesions that enable abnormalized vessels to facilitate cancer aggressiveness. Targeting this type of adhesion complex could be a safe and effective therapeutic option to suppress cancer progression.

## Introduction

Development and maintenance of the body’s organs require an adequate blood supply to bring oxygen and nutrients to the tissues (1). Tumor growth also depends on formation of new blood vessels from existing ones. However, hastily built new vessels tend to be leaky and functionally immature; these are referred to as “abnormalized” (2). Impaired perfusion and oxygenation caused by vessel abnormalization activates a metabolic switch in cancer cells and promotes focal invasion and distant metastasis (2).

VEGF is the most potent angiogenic factor that drives tumor angiogenesis. VEGF inhibitors are used widely to treat various types of human cancers (2–4). Although VEGF inhibitors suppress tumor progression, the therapeutic effects are sometimes limited; paradoxically, VEGF inhibitors can promote aggressiveness by increasing tumor hypoxia or the number of abnormalized vessels (5–8). Moreover, damage to normal vessels can cause severe adverse effects, including cerebral hemorrhage and intestinal perforation (9, 10). In light of these clinical situations, it will be important to identify drugs that target abnormalized but not normal blood vessels, which could be used in combination with immunotherapeutic drugs (11, 12) to combat cancer.

Fibronectin leucine-rich transmembrane protein 2 (FLRT2), a member of the FLRT family of proteins, acts as a repulsive ligand of the UNC5 receptor family; it was initially identified as a chemorepellent in neurons (13–15). Outside of the nervous system, FLRT2 is expressed in endothelial cells in the placental labyrinth, and FLRT2 contributes to its proper formation (16). It was also reported that FLRT2 possesses dual functions: heterophilic binding to UNC5B, which causes cellular repulsion, and homophilic binding, which supports intercellular adhesion (15). FLRT proteins also interact with adhesion GPCRs of the latrophilin family (17), with established functions in brain development.

Interendothelial junctions, which are essential for vascular integrity and function, comprise tight junctions, adherens junctions, and a variety of other adhesion molecules such as PECAM1 and angiopoietins (18). These junctions maintain a stable vascular system and control vascular permeability. In tumors, loss of interendothelial adhesion is a hallmark of abnormalized vessels, which promote cancer progression by enabling transvascular migration of tumor cells (2, 18, 19).

Here, we analyzed human colorectal cancer samples and found that FLRT2 is expressed abundantly in endothelial cells lining abnormalized vessels. Deletion of endothelial *Flrt2* in mice selectively ablated these abnormalized vessels, thereby suppressing tumor invasion and metastasis without triggering the metabolic switch. Mechanistically, Flrt2 facilitates interendothelial adhesion by forming homophilic bonds, thereby protecting cells from oxidative stress. Taken together, these findings suggest that abnormalized vessels exploit noncanonical interendothelial adhesions, which are different from the canonical junctions in healthy vessels, to disseminate tumor cells.

## Results

### FLRT2 is expressed in the tumor endothelial cells of advanced human colorectal cancers.

**Figure 1:**
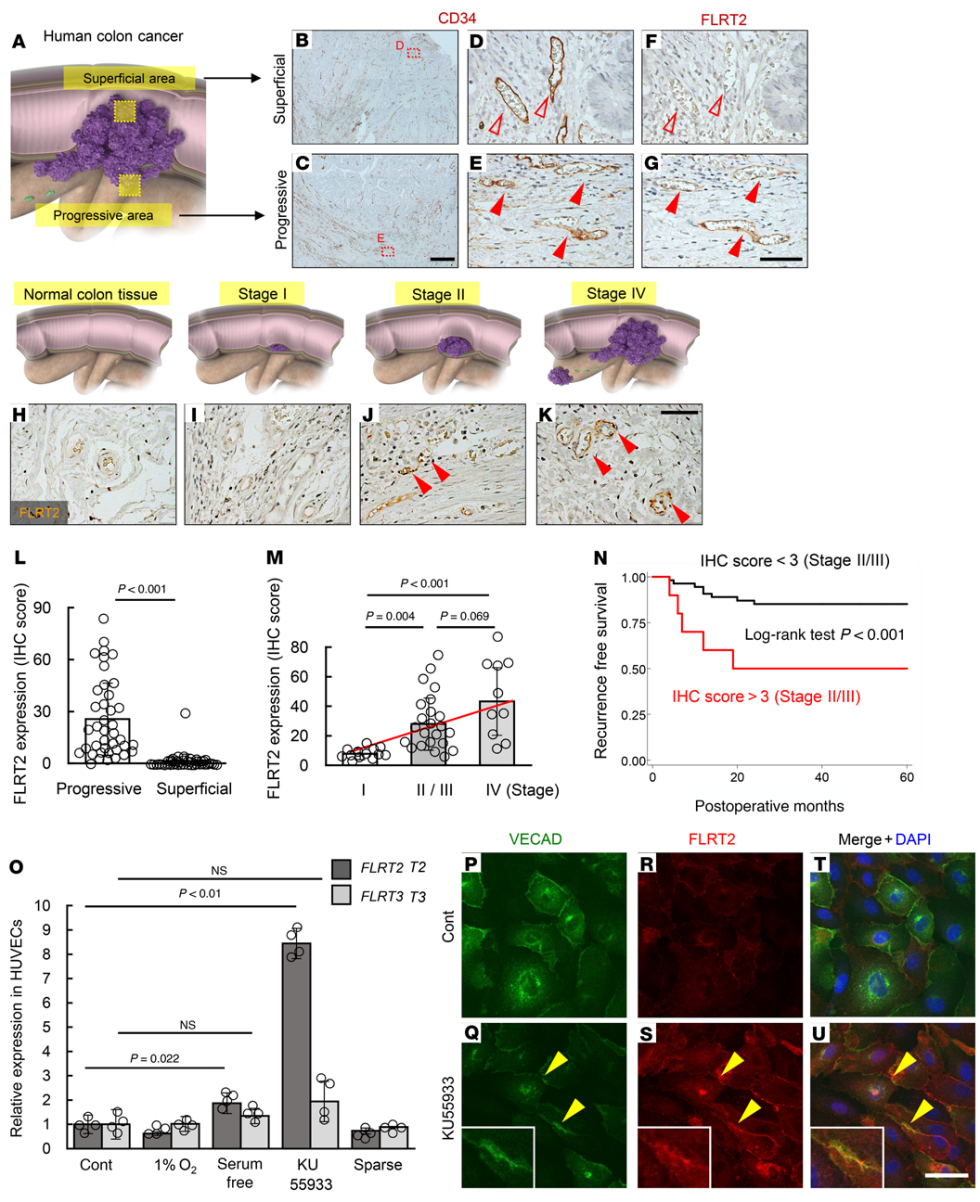
Endothelial expression of FLRT2 in advanced human colorectal cancers. (**A**) Schematic diagram depicting the location of the progressive and superficial areas in human colorectal cancer. (**B**–**G**) Immunohistochemical analysis of FLRT2 and CD34 expression in serial sections of paraffin-embedded samples cut from resected tumors from stage IV cases. Endothelial cells in the progressive area (closed arrowheads), but not those in the superficial area (open arrowheads), express FLRT2. (**H**–**K**) Immunohistochemical analysis of FLRT2 expression in the progressive area of tumors from individuals with various stages of colorectal cancer. Endothelial cells in tumors at advanced stages show FLRT2 expression (arrowheads). (**L**) Quantification of FLRT2 expression, as measured by the IHC score (*n =* 46 each). (**M**) Quantification of FLRT2 expression in the progressive area of tumors at various stages (*n =* 13 [stage I], 23 [stage II/III], 10 [stage IV]). The red line represents regression. (**N**) Kaplan-Meier curve showing recurrence-free survival of patients with stage II or III colorectal cancer, stratified according to FLRT2 expression (high- and low-scoring tumors). The log-rank test was used to compare differences between groups (*n =* 66). (**O**) Relative expression of FLRT2 and FLRT3 under various culture conditions (*n =* 4 each). (**P**–**U**) Immunocytochemistry analysis of HUVECs. Localization of FLRT2 proteins at the intracellular junctions (arrowheads) increased markedly after oxidative stress induced by KU55933. Representative images for 3 independent experiments are shown. Scale bars: 500 μm (**B** and **C**); 50 μm (**D**–**K** and **P**–**U**). Data are presented as the mean ± SD. Comparisons between mean values of 2 groups were evaluated using a 2-tailed Student’s *t* test. Comparisons among multiple groups (**M** and **O**) were evaluated using 2-way ANOVA followed by Bonferroni’s multiple-comparison test. Kaplan-Meier curves and the log-rank test were used to compare survival among the groups.

Previously, we showed that Flrt2 is expressed by endothelial cells in the placental labyrinth, but not in other vascular beds, during mouse embryogenesis (16). Because the placenta produces ROS and is exposed to oxidative stress (20), we sought to examine expression of FLRT2 in abnormalized tumor vessels, which are also exposed to oxidative stress (21). We examined expression of FLRT2 by immunostaining tissues from a cohort of 47 patients with colorectal cancer who had undergone surgical resection. Colorectal cancers are epithelial cell–derived malignant tumors that infiltrate the submucosal, muscularis, and serosal layers; therefore, we examined expression of FLRT2 in superficial and progressive areas (Figure 1A). Interestingly, FLRT2 was expressed abundantly in CD34^+^ endothelial cells within areas of progression in stage IV cancer samples, but far less so in superficial areas (Figure 1, B–G). Blood vessels in the progressive area were apparently angiogenic, with many structures with an appearance of sprouting (Supplemental Figure 1A; supplemental material available online with this article; https://doi.org/10.1172/JCI153626DS1). In addition, while there was no detectable expression of FLRT2 in normal colon tissues or stage I tumors, strong expression was observed in progressive areas of stage II, III, and IV tumors, which are defined as advanced cancers (Figure 1, H–M, and Supplemental Figure 1B). Because these results suggest that expression of endothelial FLRT2 is related to long-term prognosis, we examined another cohort of 66 patients who underwent curative surgical resection for pathological stage II or III colorectal cancer and were followed up for 5 years (Supplemental Figure 1C). Strikingly, Kaplan-Meier curves for recurrence-free survival (RFS) showed that patients with high expression of endothelial FLRT2 had a significantly poorer prognosis than those with low expression (Figure 1N). Furthermore, multivariate analyses using a Cox proportional hazards model revealed that high expression of FLRT2 by endothelial cells was an independent risk factor for RFS, regardless of tumor stage (Supplemental Figure 1D). Taken together, these data suggest that expression of FLRT2 is an independent biomarker for predicting prognosis of colorectal cancers (in addition to canonical staging). Next, we asked what triggers expression of FLRT2 by endothelial cells. In agreement with a previous report (22), we found that VEGF upregulated expression of FLRT3 (another member of the FLRT family), but not FLRT2, in cultured human umbilical vein endothelial cells (HUVECs; Supplemental Figure 2A). Among other stimuli, we found that elevation of ROS mediated by KU55933, an ATM inhibitor (21), or treatment with H_2_O_2_ significantly increased expression of FLRT2 by HUVECs (Figure 1O and Supplemental Figure 2B). Increased expression of FLRT2 protein was localized to intercellular junctions as well as the cytoplasm (Figure 1, P–U). Flrt2 was also abundantly expressed in aberrantly expanding neovessels, which are known to be exposed to high oxidative stress (21), in ischemic retinopathy but not in surrounding stable vessels (Supplemental Figure 2, C and D). This expression of FLRT2 was suppressed by administration of the antioxidant *N*-acetyl-L-cysteine (Supplemental Figure 2, C–F). In combination with the clinical data, these data indicate that FLRT2 is expressed by endothelial cells lining tumor vessels that are exposed to high levels of oxidative stress.

### Endothelial Flrt2 sustains abnormalized tumor vessels in mice.

**Figure 2:**
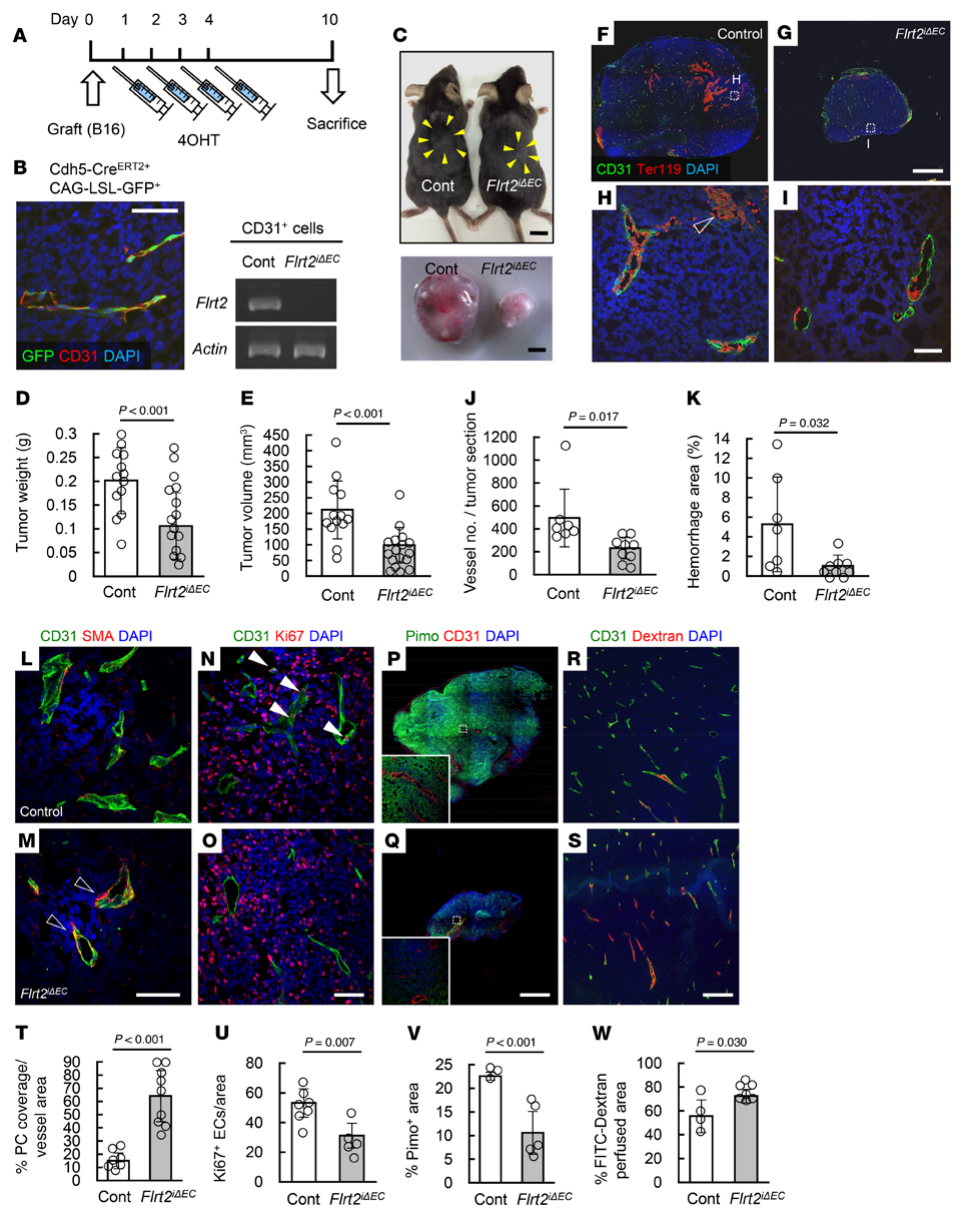
Endothelial Flrt2 sustains abnormalized tumor vessels in mice. (**A**) Protocol for 4OHT injections and tumor inoculations. (**B**) Representative IHC of B16 tumors on day 10 (*n =* 3) and representative PCR results (*n =* 3) showing expression of *Flrt2* in isolated CD31^+^ tumor endothelial cells. (**C**) Tumors resected from mice 10 days after transplantation of B16 cells into the back skin (arrowheads). (**D** and **E**) Measurement of tumor weight and volume (*n* = 13 [control], 15 [Flrt2^iΔEC^]). (**F**–**K**) Immunohistochemical analysis of tumor sections (*n* = 7 [control], 9 [Flrt2^iΔEC^]). Intratumoral hemorrhage (open arrowheads) in tumors from control mice is greater than that in *Flrt2*^iΔEC^ mice. (**L**–**W**) Immunohistochemical analysis of tumor sections (*n* = 7, 9, 7, and 5 [control], 3, 5, 4, and 7 [Flrt2^iΔEC^]). Tumors from *Flrt2*^iΔEC^ mice show reduced endothelial proliferation (closed arrowheads) and increased pericyte coverage (open arrowheads). Scale bars: 1 cm (**C** [top]); 2 mm (**C** [bottom], **F**, **G**, **P**, and **Q**); 200 μm (**R** and **S**); 50 μm (**B**, **H**, **I**, and **L**–**O**). Data are presented as the mean ± SD. Comparisons between mean values of 2 groups were evaluated using a 2-tailed Student’s *t* test.

**Figure 3:**
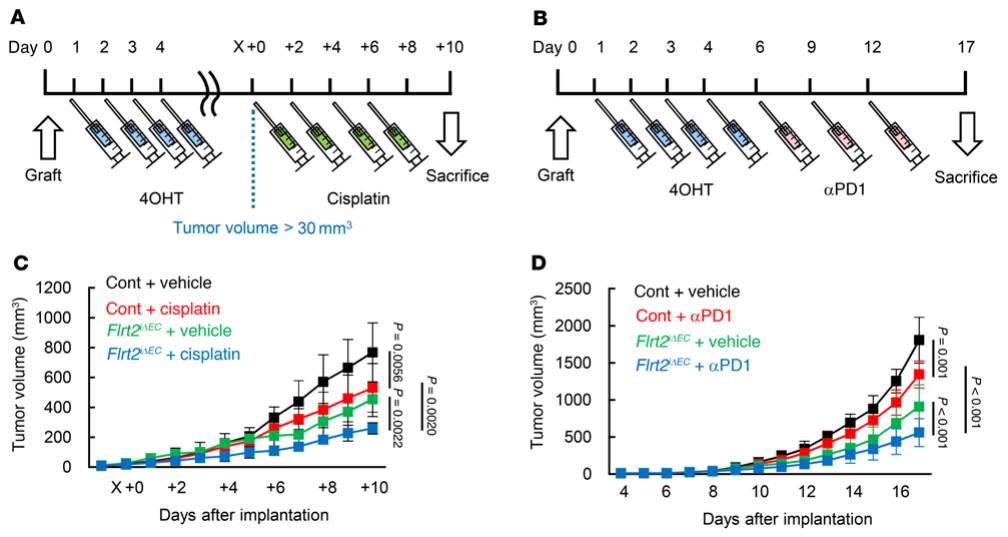
Endothelial Flrt2 deletion potentiates tumor-suppressing effects of cytotoxic and immunotherapeutic drugs. (**A**) Protocol for injection of 4OHT and cisplatin treatment (2.5 mg/kg) and for tumor inoculation. (**B**) Protocol for injection of 4OHT, anti-PD1 antibody treatment, and tumor inoculation. (**C**) Measurement of tumor volume (*n* = 14 [control+vehicle], 11 [control+cisplatin], 8 [Flrt2^iΔEC^+vehicle], 10 [Flrt2^iΔEC^+cisplatin]). (**D**) Measurement of tumor volume (*n* = 10 [control+vehicle], 10 [control+cisplatin], 9 [Flrt2^iΔEC^+vehicle], 11 [Flrt2^iΔEC^+cisplatin]). Data are presented as the mean ± SD. Comparisons among multiple groups were evaluated using 2-way ANOVA followed by Bonferroni’s multiple-comparison test.

Next, we used a syngeneic murine B16 melanoma model to evaluate the bona fide role of endothelial FLRT2 in tumor progression. Because conventional and endothelial cell–specific *Flrt2*-knockout mice are embryonically lethal (16, 23), we generated tamoxifen-inducible endothelial cell–specific *Flrt2*-knockout mice (*Cdh5-BAC-Cre^ERT2^Flrt2^fl/fl^* mice, referred to hereafter as *Flrt2*^iΔEC^ mice). We subcutaneously implanted these mice with B16 cells and then injected them with 4-hydroxytamoxifen (4OHT) for 4 consecutive days (Figure 2A). This protocol effectively deleted *Flrt2* from the tumor endothelial cells (Figure 2B). At 10 days after implantation, tumor growth was moderately suppressed in *Flrt2*^iΔEC^ mice (Figure 2, C–E). Histologically, *Flrt2*^iΔEC^ mice showed a moderate reduction in the total number of tumor vessels and a marked reduction in the area of intratumoral hemorrhage (Figure 2, F–K). Considering intratumor variability of the number of blood vessels (24), we counted all of the vessels in the entire field within the plane of maximum cut of tumor samples. The percentage of blood vessels covered by smooth muscle actin^+^ (SMA^+^) mural cells (25), namely stable vessels, was markedly increased in *Flrt2*^iΔEC^ mice (Figure 2, L, M, and T). Endothelial proliferation in *Flrt2*^iΔEC^ mice was lower than that in control mice (Figure 2, N, O, and U). Tumors in *Flrt2*^iΔEC^ mice showed reduced intratumor hypoxia, accompanied by increased vessel perfusion (Figure 2, P–S, V, and W). Intracardiac injection of 10 kDa dextran showed decreased vessel leakiness in *Flrt2*^iΔEC^ mice (Supplemental Figure 3, A and B), supporting the reduced hemorrhaging data. Accumulation of macrophages in the hemorrhagic lesion was not apparent (Supplemental Figure 3, C and D). Healthy vessels outside the tumor, postnatal retinal vascularization, and wound healing were not affected by *Flrt2* deletion (Supplemental Figure 4, A–H). Similar to the transient effect of VEGF blockade during the ‘‘normalization window’’ (26, 27), deletion of endothelial *Flrt2* increased the tumor-suppressing effects of a cytotoxic agent, cisplatin (28, 29), and those of immunotherapeutic drugs (anti-PD1 antibodies) (Figure 3). Next, we injected tamoxifen 14 days after transplantation and examined the immediate effect of intermittent FLRT2 deletion, which revealed ablation of abnormalized vessels and a reduction in hypoxia in *Flrt2*^iΔEC^ mice as in the primary protocol for tamoxifen (Supplemental Figure 5). These data suggest that FLRT2 indeed functions in vessel maintenance.

### Deletion of endothelial Flrt2 induces oxygen-glucose uncoupling in tumors.

**Figure 4:**
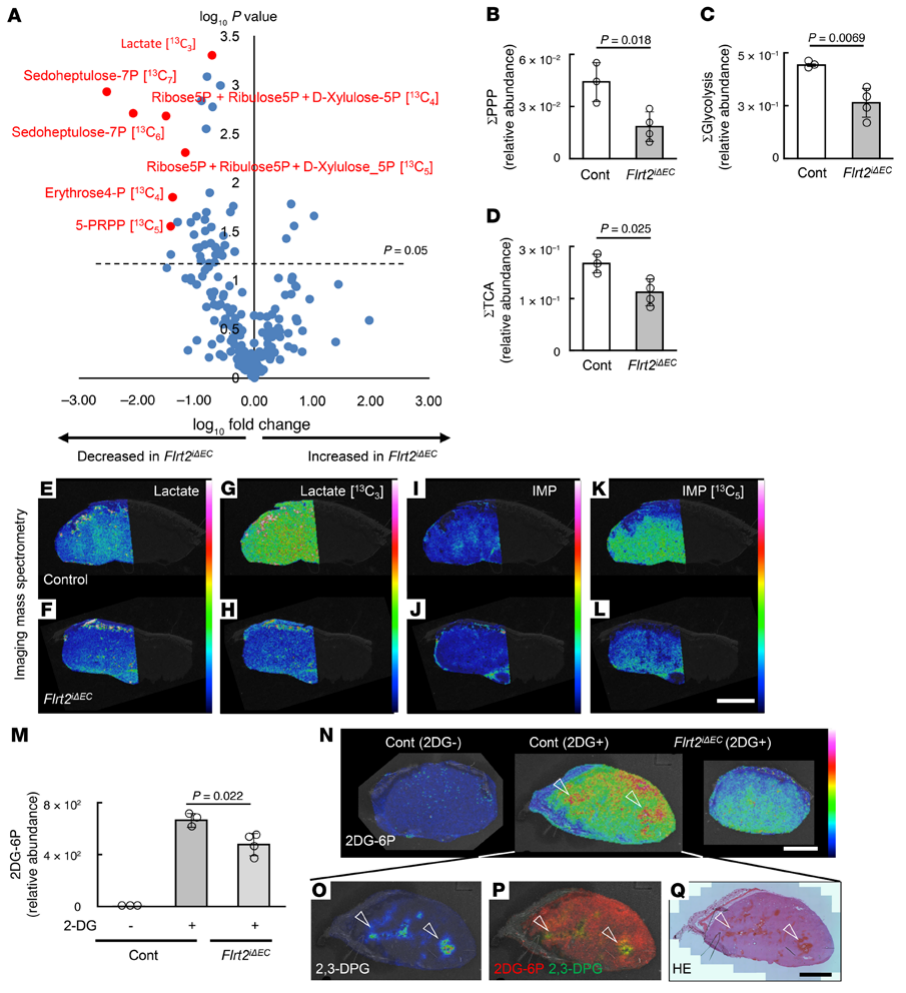
Deletion of endothelial Flrt2 induces oxygen-glucose uncoupling in tumors. (**A**) Volcano plot showing the amount of metabolites in *Flrt2*^iΔEC^ mice relative to that in control mice. The red dots represent ^13^C-labeled metabolites of the pentose phosphate pathway (PPP), which were significantly reduced in *Flrt2*^iΔEC^ mice. The horizontal dotted line represents a *P* value of 0.05. Mice were pretreated with ^13^C_6_-glucose to reveal differences in glucose metabolism pathways within the tumor. (**B**–**D**) Relative intracellular levels of components of PPP, the glycolytic system, and the TCA cycle, as measured by IC-MS analysis (peak area/internal standard/tissue mg) (*n* = 3 [control], 4 [Flrt2^iΔEC^]). (**E**–**L**) Representative images showing intratumoral distribution of glucose metabolites, as assessed by imaging mass spectrometry (IMS). Representative images for 3 independent experiments are shown. (**M**) Amount of 2-deoxy-D-glucose (2-DG) leaking from blood vessels and accumulating in tumor cells, as quantified by IC-MS analysis (*n* = 3 [control with 2-DG], 3 [control without 2-DG], 3 [Flrt2^iΔEC^]). 2-DG is converted to 2DG-6P intracellularly and then accumulates in the tumor (see Supplemental Figure 7). (**N**–**Q**) IMS showing the amount and localization of 2DG-6P and 2,3-DPG. Arrowheads indicate colocalization of 2DG-6P and 2,3-DPG in the hemorrhagic area. Representative images for 3 independent experiments are shown. Scale bars: 2 mm (**E**–**L** and **N**–**Q**). Data are presented as the mean ± SD. Comparisons between mean values of 2 groups were evaluated using a 2-tailed Student’s *t* test. IMP, inosine monophosphate.

Next, we conducted a metabolomic analysis to better understand how tumors respond to deletion of endothelial *Flrt2*. Reprogramming of glucose metabolism in cancer cells accelerates not only aerobic glycolysis but also anabolic pathways that generate macromolecules (e.g., nucleotides) via the pentose phosphate pathway (PPP) and glycogens synthesis via the uridine diphosphate/glucose pathway, thereby driving tumor progression (30). Moreover, vessel leakiness is supposed to affect glucose uptake of tumor cells, particularly in highly glycolytic tumors such as B16 melanoma. Therefore, we injected mice intraperitoneally with full-labeled glucose ([U-^13^C_6_] glucose) and traced the metabolic fate of ^13^C_6_ glucose–derived carbon atoms. Volcano plot analysis shows that the flow toward the PPP was reduced significantly in *Flrt2*^iΔEC^ mice (Figure 4, A and B, and Supplemental Figure 6); the ratio of the reduction was greater than that for glycolysis (Figure 4C) and for the TCA cycle (Figure 4D). Imaging mass spectrometry (IMS) revealed reductions in key metabolites, namely lactate (a glycolytic end product) and inosine monophosphate (an intermediate in the PPP) (Figure 4, E–L). To evaluate glucose utilization by tumor cells, we injected mice intraperitoneally with 2-deoxy-D-glucose (2-DG), which is taken up by cells and converted into a phosphorylated form (2DG-6-phosphate [2DG-6P]) that cannot undergo further downstream glycolysis (31). The amount of 2DG-6P in the tissues of *Flrt2*^iΔEC^ mice was reduced significantly (Figure 4, M and N, and Supplemental Figure 7). Moreover, when we checked the distribution of 2,3-DPG, a metabolite that is enriched in erythrocytes (32), we found that most of the 2DG-6P was localized in the 2,3-DPG^+^ hemorrhagic area (Figure 4, O–Q), suggesting that the injected 2-DG leaked out of abnormalized vessels in control mice but not in *Flrt2*^iΔEC^ mice. These data indicate that the reduction in abnormalized vessels in *Flrt2*^iΔEC^ mice increased oxygen provision and decreased glucose leakage and that this oxygen-glucose uncoupling contributes to the suppression of not only aerobic glycolysis, but also anabolic pathways.

### Deletion of Flrt2 prevents tumor metastases and spontaneous tumor formation.

**Figure 5:**
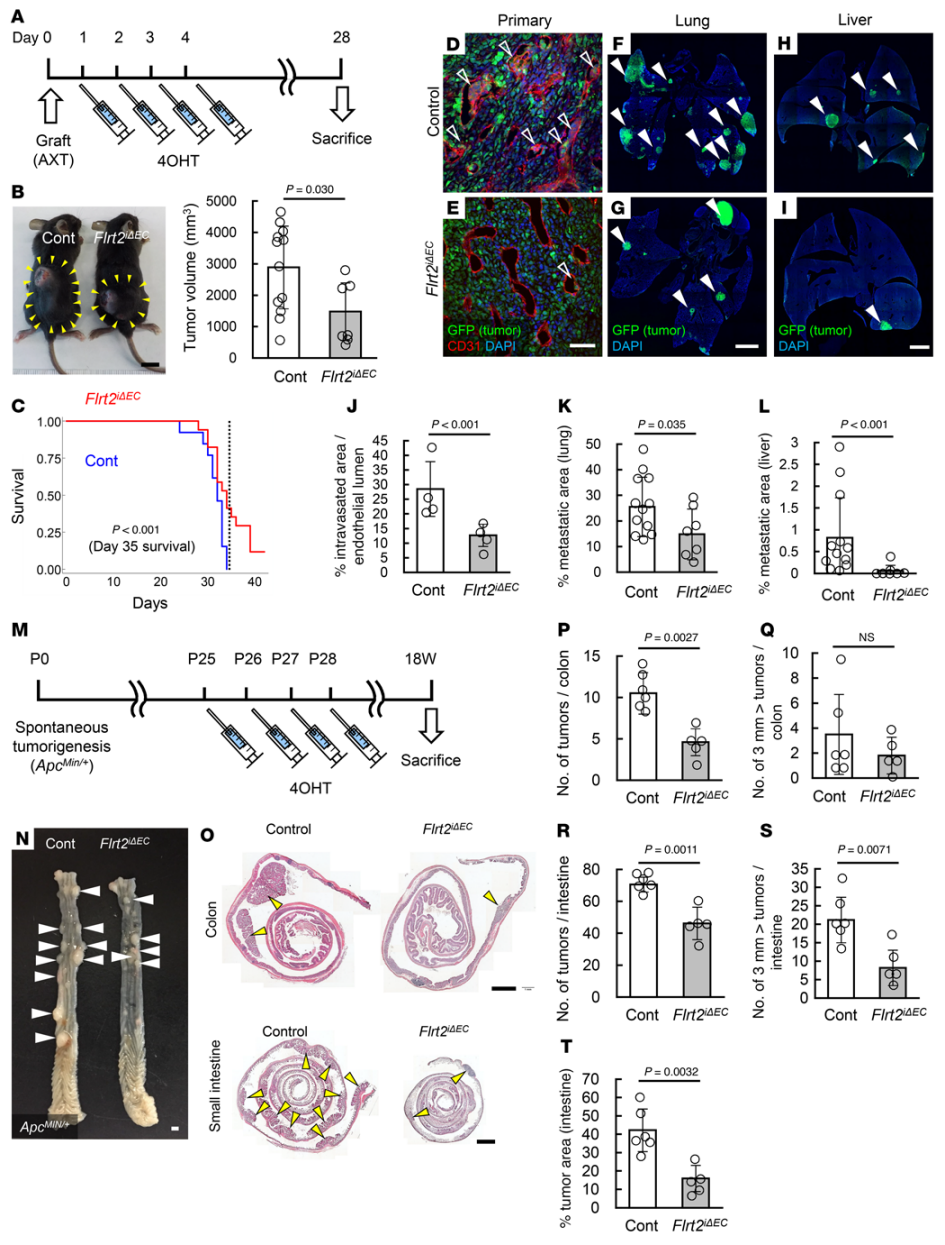
Deletion of Flrt2 prevents tumor metastases and spontaneous tumor formation. (**A**) Protocol for 4OHT injections and tumor inoculation. (**B**) Measurement of tumor volume in mice 28 days after transplantation of AXT cells into the back skin (arrowheads) (*n* = 12 [control], 7 [Flrt2^iΔEC^]). (**C**) Kaplan-Meier survival analysis of mice inoculated subcutaneously with AXT cells (*n =* 13 [control], 17 [Flrt2^iΔEC^]). (**D**–**L**) Immunohistochemical analysis of primary tumors, lungs, or livers from mice 28 days after transplantation of AXT cells into the back skin (*n* = 4 [control primary], 4 [control lung], 12 [control liver], 7 [Flrt2^iΔEC^ primary], 12 [Flrt2^iΔEC^ lung], 7 [Flrt2^iΔEC^ liver]). Intravasation of tumor cells (open arrowheads) in primary tumors and their metastasis to distant organs (closed arrowheads) are less pronounced in *Flrt2*^iΔEC^ mice. (**M**) Protocol for tamoxifen injection into *APC^Min/+^* model mice. (**N**–**T**) Representative images showing the macroscopic appearance of the colon, along with the sectioned colon and small intestine specimens stained with H&E (*n =* 6 [control], 5 [Flrt2^iΔEC^]). *APC^Min/+^Flrt2*^iΔEC^ mice have fewer and smaller tumors (arrowheads) in both the colon and small intestine than *APC^Min/+^* mice. Scale bars: 1 cm (**B**); 2 mm (**F**–**I**, **N**, and **O**); 50 μm (**D** and **E**). Data are presented as the mean ± SD. Comparisons between mean values of 2 groups were evaluated using a 2-tailed Student’s *t* test. Kaplan-Meier curves and the log-rank test were used to compare survival among the groups.

The data above indicate that the deletion of endothelial *Flrt2* efficiently suppresses tumor angiogenesis without triggering metabolic reprogramming. Thus, we utilized a syngeneic mouse cancer model based on AXT cells, a highly metastatic osteosarcoma cell line (33). Growth of primary tumors in the back skin of *Flrt2*^iΔEC^ mice was slightly inhibited (Figure 5, A and B). Kaplan-Meier curves for overall survival showed that the 35 day overall survival was significantly higher for *Flrt2*^iΔEC^ mice than for control mice (Figure 5C). Tumors in *Flrt2*^iΔEC^ mice showed reduced intratumor hypoxia (Supplemental Figure 8A). Accordingly, intravasation of tumor cells derived from primary tumors and resultant metastasis to the liver and lungs were markedly lower in *Flrt2*^iΔEC^ mice (Figure 5, D–L, and Supplemental Figure 8B). To test the contribution of Flrt2 in the premetastatic niche, we examined the expression of Flrt2 in endothelial cells before and after transplantation of AXT cells. We did not observe any apparent changes in the expression of Flrt2 (Supplemental Figure 8C). The metastasis phenotype of *Flrt2*^iΔEC^ mice was in stark contrast to that of endothelial *Vegfr2*-knockout mice (*Cdh5-BAC-Cre^ERT2^Vegfr2^fl/fl^*, referred to hereafter as *Vegfr2*^iΔEC^ mice); AXT cells transplanted into *Vegfr2*^iΔEC^ mice showed increased metastasis to the liver, despite the decreased growth of primary tumors, likely due to the increase in hypoxia caused by excessive destruction of tumor vessels (Supplemental Figure 9). The expression of endothelial Flrt2 in B16 tumors transplanted into *Vegfr2*^iΔEC^ mice was not impaired (Supplemental Figure 10), in agreement with the in vitro data showing that VEGF signaling is not related to the expression of FLRT2 (Supplemental Figure 2A). From the viewpoint of actual human cancers, there is a need to study more clinically relevant models that recapitulate modes of cancer progression, such as spontaneous tumorigenesis (34, 35). Therefore, we utilized *APC^Min/+^* mice (36). At 18 weeks of age, *APC^Min/+^Flrt2*^iΔEC^ mice developed fewer tumors in both the colon and small intestine than *APC^Min/+^* mice (Figure 5, M–T). The number of tumors greater than 3 mm in diameter in the small intestine of *APC^Min/+^Flrt2*^iΔEC^ mice was significantly lower than that in *APC^Min/+^* mice (Figure 5, Q and S).

### Intercellular homophilic binding of FLRT2 protects ECs from oxidative stress.

**Figure 6:**
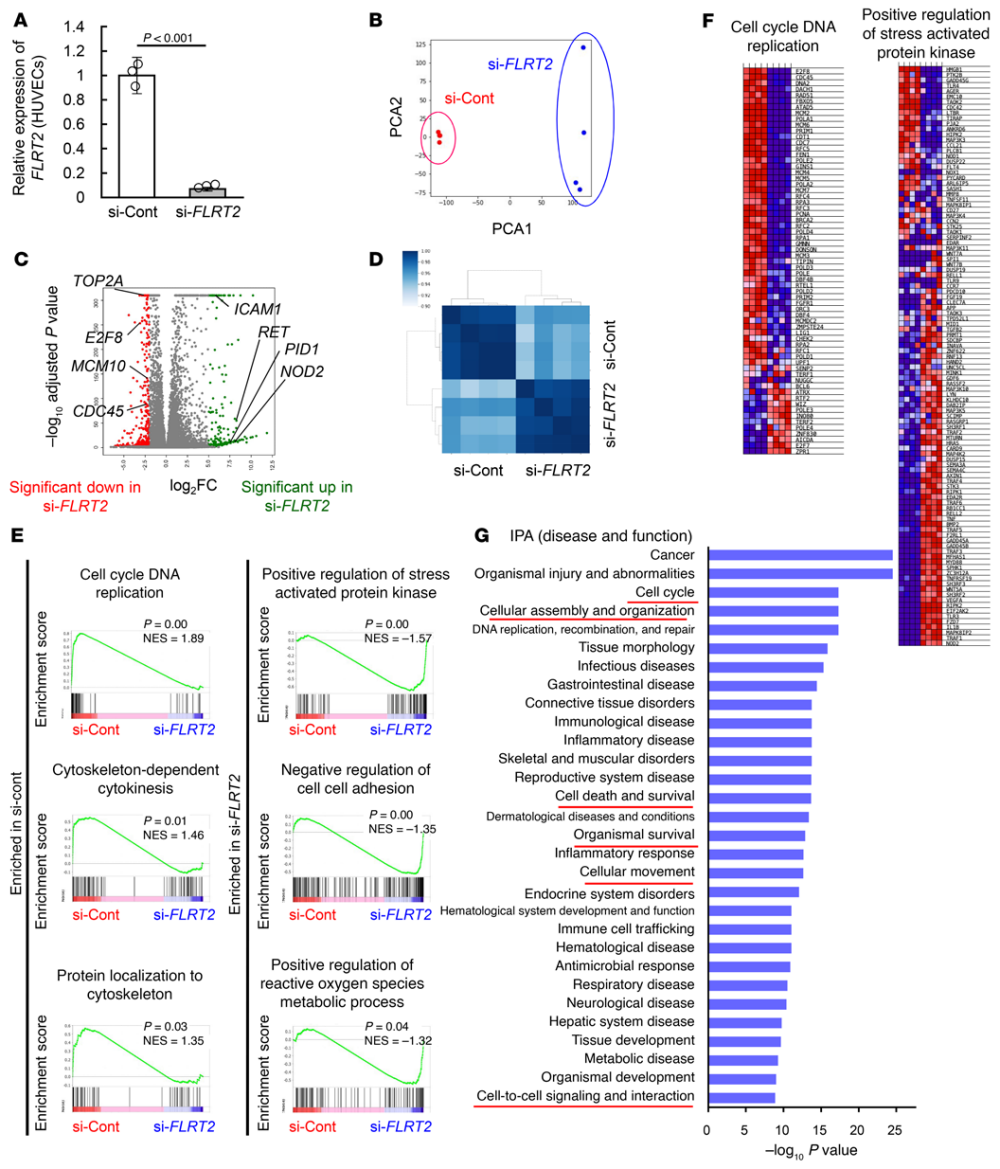
FLRT2 silencing affects genes related to oxidative stress and intercellular adhesion. (**A**) RT-PCR analysis of HUVECs treated with siRNA (*n =* 3 each). (**B**) Principal component analysis of RNA-Seq data from HUVECs treated with si-Cont or si-*FLRT2*. (**C**) Volcano plot of genes differentially expressed by HUVECs treated with si-Cont or si-*FLRT2*. Genes significantly upregulated or downregulated (*q* < 0.01, logFC > 5 or < –2) in si-*FLRT2*–treated HUVECs are marked in red or green, respectively. (**D**) Correlation matrix showing the Pearson correlation coefficients for each RNA-Seq experiment. (**E** and **F**) GSEA plots and heat maps of up- or downregulated genes in si-*FLRT2*–treated HUVECs. (**G**) Ingenuity Pathway Analysis (IPA) of differentially expressed genes between HUVECs treated with si-Cont or si-*FLRT2*. Terms underscored by red lines are related to the phenotype in *Flrt2*^iΔEC^ mice. Data are presented as the mean ± SD. Comparisons between mean values of 2 groups were evaluated using a 2-tailed Student’s *t* test.

**Figure 7:**
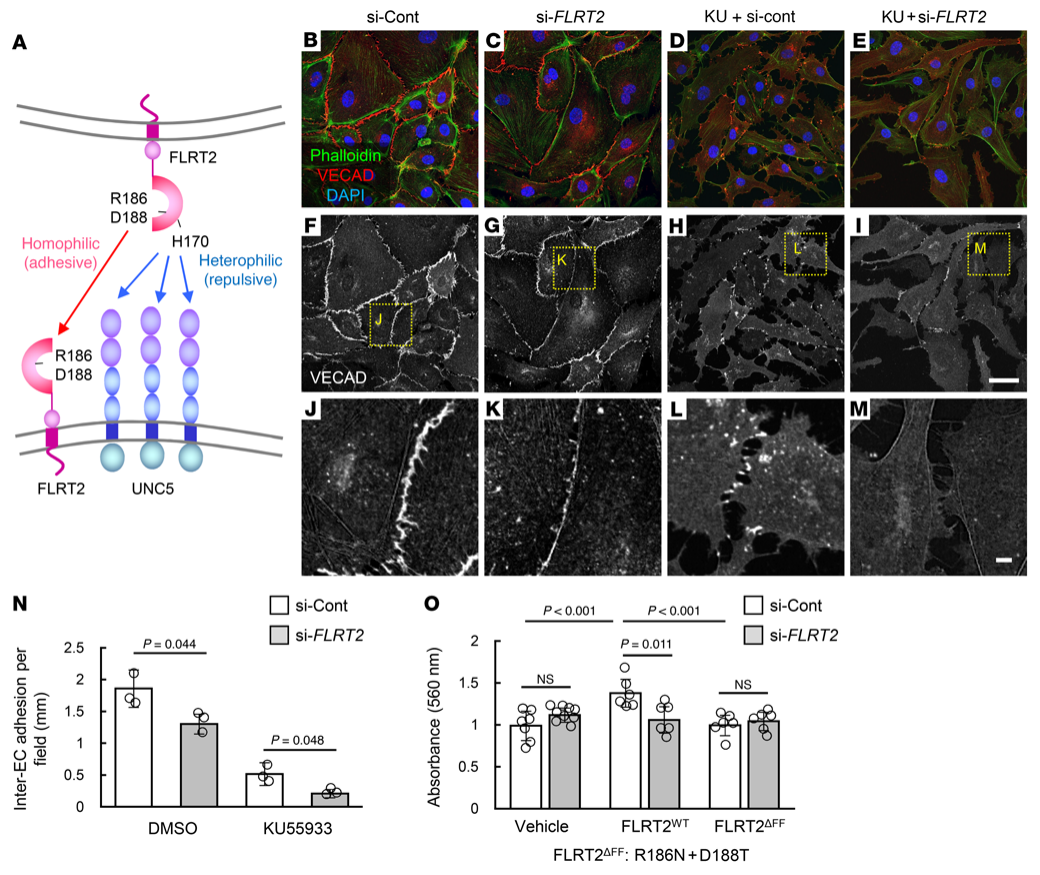
Intercellular adhesion via homophilic binding of FLRT2 protects ECs against oxidative stress. (**A**) A scheme depicting the wide-ranging actions of FLRT2. (**B**–**N**) Immunocytochemical analysis of HUVECs, and analysis of VECAD^+^ interendothelial cell (EC) adhesion (*n =* 3 each). (**O**) Adhesion of EC harboring recombinant FLRT2 proteins (*n =* 7 [Vehicle si-Cont], 8 [Vehicle si-FLRT2], 6 [FLRT2WT si-Cont], 6 [FLRT2WT si-FLRT2], 6 [FLRT2ΔFF si-Cont], 6 [FLRT2ΔFF si-FLRT2]). Data are presented as the mean ± SD. Scale bar: 50 μm (**B**–**I**); 5 μm (**J**–**M**). Comparisons between mean values of 2 groups were evaluated using a 2-tailed Student’s *t* test. Comparisons among multiple groups (**O**) were evaluated using 2-way ANOVA followed by Bonferroni’s multiple-comparison test.

Because Flrt2 acts primarily as a repulsive ligand for the Unc5b receptor (14, 16), we injected endothelial cell–specific *Unc5b*-knockout mice (*Cdh5-BAC-Cre^ERT2^Unc5b^fl/fl^*; referred to hereafter as *Unc5b*^iΔEC^ mice) with B16 melanoma cells. Unexpectedly, tumor growth, vascularization, and intratumor hemorrhage were not altered significantly in *Unc5b*^iΔEC^ mice (Supplemental Figure 11, A–E). Therefore, to explore alternative mechanisms underlying the vascular normalizing effect of *Flrt2* deletion, we performed comparative transcriptomics analyses using HUVECs subjected to si-RNA–mediated knockdown of *FLRT2*. Principal components analyses and Pearson correlation matrix analysis of RNA-Seq data revealed global changes in the transcriptome of HUVECs treated with the si-Control or si-*FLRT2* (Figure 6, A–D). Extraction of differentially expressed genes (DEGs) and gene set enrichment analysis (GSEA) indicated that the genes most affected by si-*FLRT2* were associated with immune responses represented by type I interferon signaling, responses to viruses, and peptide antigen binding (Supplemental Figure 12A and Supplemental Table 1), none of which are relevant to angiogenesis. Therefore, we checked subleading DEGs and ontologies and found that si-*FLRT2* downregulated genes related to the cytoskeleton (*RHOB*, *RASA1*, *ROCK1*, etc.) and cell cycle (*CDC45*, *E2F8*, *MCM10*, etc.), whereas upregulated genes were related to oxidative stress (*PID1*, *ICAM1*, *PTX3*, etc.) and intercellular adhesion (*IDO1*, *CECAM1*, *LGALS9*, etc,) (Figure 6, C and E–G). We next examined the expression of UNC5 receptors by checking their absolute mRNA amounts (transcripts per kilobase million) in HUVECs. Although the UNC5A, -C, and -D expression was nearly undetectable, UNC5B expression was abundant and was significantly increased by treatment with si-*FLRT2* (Supplemental Figure 12B). Considering that FLRT2 can support intercellular adhesion through homophilic binding (Figure 7A) and accumulate at cell-to-cell junctions under the control of ROS (Figure 1, P–U), we suspected that homophilic FLRT2 binding acts adhesively, particularly when ROS are elevated. Treatment of HUVECs with si-*FLRT2* alone led to a significant reduction in the number of adherens junctions (Figure 7, B, C, F, G, J, K, and N). This effect of si-*FLRT2* was more prominent when intracellular ROS were elevated by KU55933 (Figure 7, B–N). To evaluate the homophilic interaction between FLRT2 molecules more closely, we utilized a synthetic nonhomophilic-binding FLRT2 mutant (FLRT2^R186N+D188T^; referred to hereafter as FLRT2^ΔFF^), created by introducing obstacle N-linked glycosylation sites (15). Although wild-type FLRT2 proteins promoted adhesion of HUVECs, FLRT2^ΔFF^ proteins did not (Figure 7O). FLRT2^ΔFF^ proteins also lacked binding to latrophilins, G protein–coupled receptors, suggesting a potential role for latrophilins in the adhesive activity of FLRT2 (37). However, adhesion to wild-type FLRT2 proteins in HUVECs was abolished by knocking down endogenous *FLRT2*, suggesting that this activity depends on homophilic binding of FLRT2 molecules (Figure 7O). Taken together, these data suggest that homophilic binding of FLRT2 supports interendothelial adhesion.

### Homophilic binding of FLRT2 constitutes tumor-specific interendothelial adhesion.

**Figure 8:**
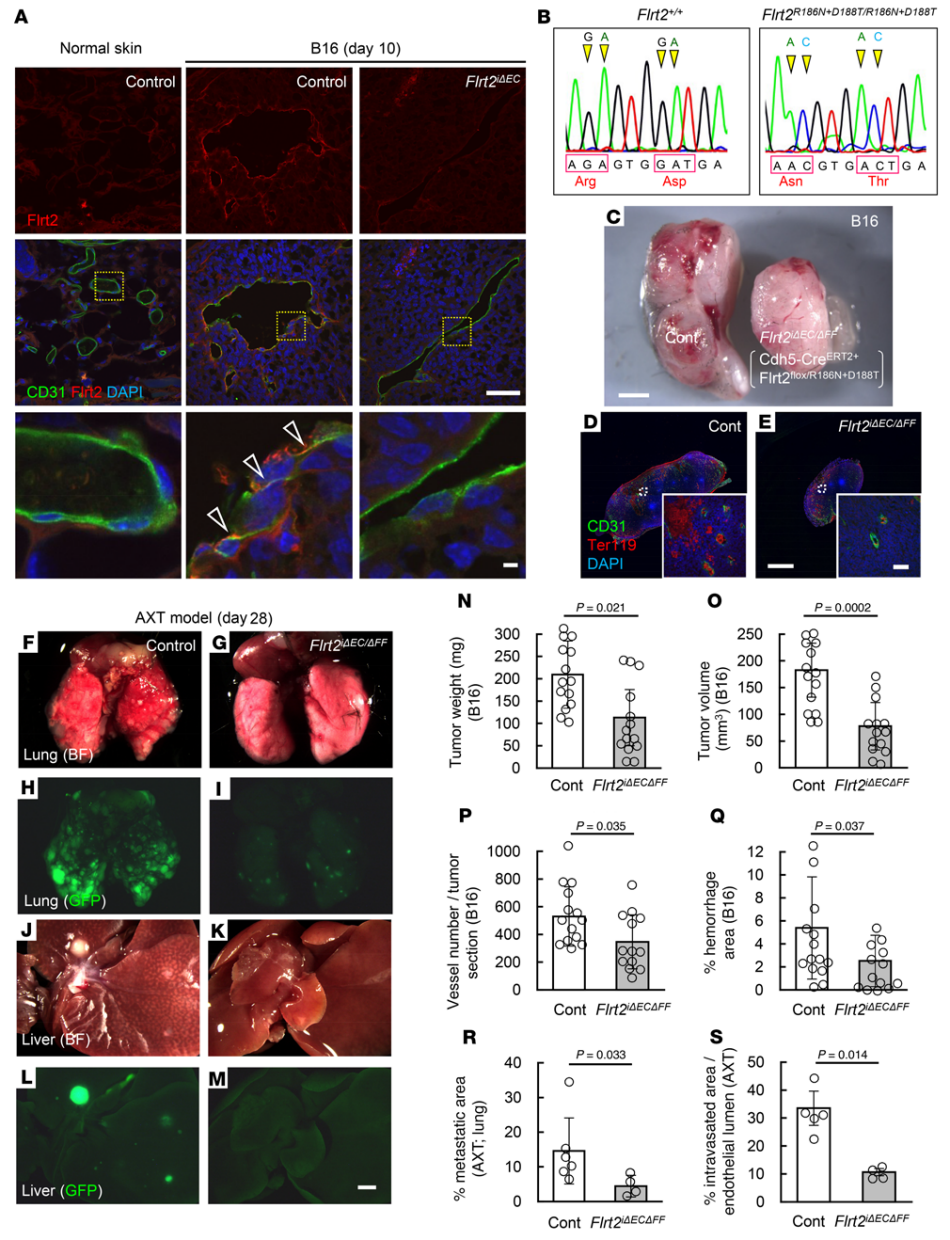
Homophilic binding of FLRT2 constitutes tumor-specific interendothelial adhesion. (**A**) Immunohistochemical analysis of sectioned tumors from mice 10 days after transplantation of B16 cells into the back skin. Arrowheads indicate interendothelial localization of FLRT2 proteins. Representative images for 3 independent experiments are shown. (**B**) Nucleotide sequences showing mutations in the *Flrt2^R186N+D188T^* allele. Representative chromatograms for 3 independent experiments are shown. (**C**–**E**) Macroscopic view and immunohistochemical analysis of sectioned tumors from mice 10 days after transplantation of B16 cells into the back skin. (**F**–**M**) Macroscopic appearance of lungs and livers from mice 28 days after transplantation of AXT cells into the back skin. (**N**–**Q**) Quantification for mice 10 days after transplantation of AXT cells into the back skin (*n =* 14 each). (**R** and **S**) Quantification for mice 28 days after transplantation of AXT cells into the back skin (*n =* 6 [control R], 4 [Flrt2iΔEC R], 5 [control S], 4 [Flrt2iΔEC S]). Data are presented as the mean ± SD. Scale bar: 2 mm (**C**–**M**); 50 μm (top 2 rows in **A**, insets of **D**, and **E**); 5 μm (bottom row in **A**). Comparisons between mean values of 2 groups were evaluated using a 2-tailed Student’s *t* test.

The above in vitro data indicate that homophilic binding of Flrt2 protects tumor vessels, which are exposed to high levels of ROS (21). Indeed, we detected Flrt2 proteins in the interendothelial cell junctions of tumors but not in normal skin vessels (Figure 8A). Next, we generated knockin mice lacking homophilic binding of Flrt2 (*Flrt2^R186N+D188T^* mice) (Figure 8B). Homozygous mutant mice lacking homophilic Flrt2 (*Flrt2^R186N+D188T/R186N+D188T^*) died before birth. Mice lacking homophilic Flrt2 binding specifically in endothelial cells (*Cdh5-Cre^ERT2+^Flrt2^flox/R186N+D188T^*; referred to hereafter as *Flrt2*^iΔ*EC/*ΔFF^ mice) showed suppression of tumor growth, angiogenesis, and hemorrhaging, similar to those in *Flrt2*^iΔEC^ mice (Figure 8, C–E and N–Q). It should be noted that the FLRT2-latrophilins interaction (17, 37, 38) could account for this phenotype, in addition to its homophilic binding. A metastatic model based on AXT cells also showed that tumors in *Flrt2*^iΔ*EC/*ΔFF^ mice demonstrated markedly lower levels of intravasation and metastasis than those in control mice (Figure 8, F–M, R, and S), suggesting that homophilic binding of Flrt2 provides the interendothelial adhesion required for maintenance of abnormalized vessels.

## Discussion

**Figure 9:**
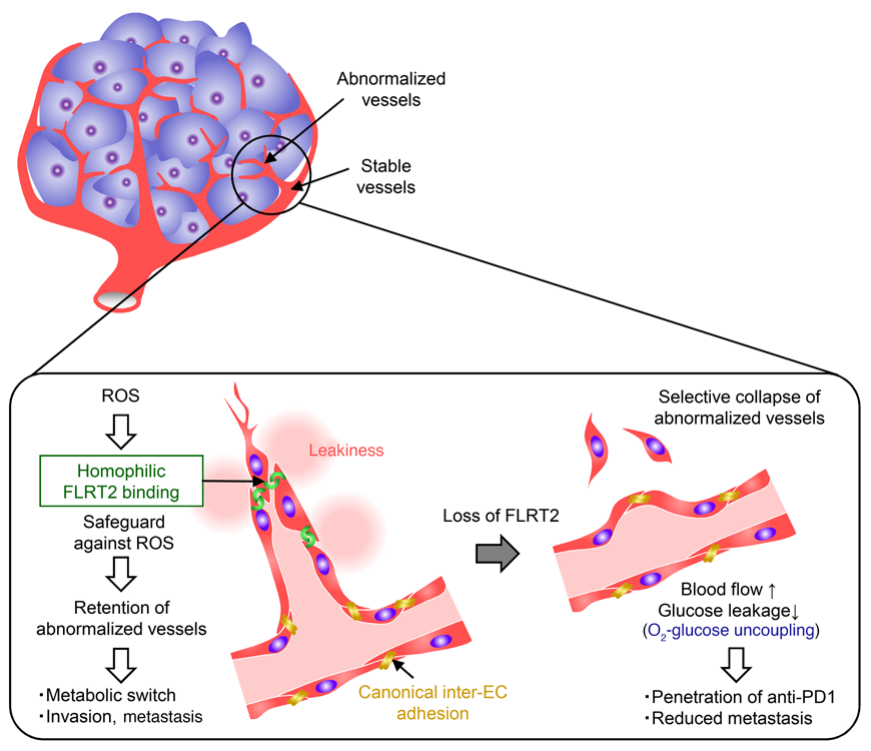
Proposed model for the role of FLRT2 in tumor angiogenesis. Proposed model for the role of FLRT2 as a safeguard against ROS in abnormalized tumor vessels, and the consequence of FLRT2 deletion.

Here, we show that noncanonical interendothelial adhesion mediated by FLRT2 supports rapidly growing abnormalized vessels in tumors, thereby protecting them from ROS-mediated damage. Deleting *Flrt2* from endothelial cells selectively pruned those abnormalized vessels, leading to uncoupled provision of oxygen and glucose and resulting in reduced tumor intravasation and metastasis without triggering metabolic reprogramming. Importantly, the relative increase in the number of mature vessels within tumors increased the efficacy of cytotoxic and immunotherapeutic drugs (Figure 9).

Our data suggest there are 2 “complementary” mechanisms of interendothelial cell adhesion. In healthy (quiescent) vessels, the canonical mechanism, regulated primarily by VE-cadherin, maintains interendothelial cell adhesion and vessel stability even in the absence of FLRT2. Downregulation of VE-cadherin is a well-known hallmark of tumor vessels, which is related to vessel abnormalization (18, 19). FLRT2, acting via a noncanonical interendothelial cell adhesion mechanism, compensates for junctional fragility caused by loss of VE-cadherin in abnormalized vessels. The phenotypes of *Flrt2*^iΔEC^ mice showed that abnormalized vessels are unable to maintain their architecture and thus collapse if both VE-cadherin and FLRT2 are absent. Oxidative stress is well known to increase internalization of VE-cadherin, resulting in an increase in vessel permeability (39). This was confirmed in our experiments using HUVECs (Figure 1, P and Q). When VE-cadherin is lost due to oxidative stress, increases in the level of FLRT2 may support the interendothelial adhesion under such a condition.

One of the most important aspects of our findings is that FLRT2 is expressed abundantly in the abnormalized vessels of advanced human colon cancers. Expression of many angiogenic molecules is regulated by oxygen, nutrients, or VEGF (1). Here, we show that expression of FLRT2 is dependent on oxidative stress but not on VEGF, a finding that agrees with that of a previous paper (22). In silico screening of human breast cancer cells showed that FLRT2 is one of the most hypermethylated and downregulated genes (40), suggesting epigenetic regulation of FLRT2 in endothelial cells. The regulatory mechanism governing expression of FLRT2, specifically the link between oxidative stress and epigenetic factors, should be comprehensively elucidated.

The opposing actions of FLRT2, repulsive (heterophilic) or adhesive (homophilic), are likely to be context dependent. Our previous in vitro experiments revealed the repulsive effects of FLRT2 when FLRT2 proteins were coated in a stripe manner (16). However, we noticed that some cells stayed on the coated stripes, whereas others migrated toward noncoated areas. These data suggest coexistence of repulsive and adhesive responses for the behavior of HUVECs on FLRT2 proteins. Our current results based on si-RNA–mediated knockdown of the *FLRT2* gene, which was not examined in the previous study, might better reflect the adhesive rather than repulsive function of FLRT2. The difference in the function of FLRT2 during development and tumor angiogenesis may be ascribed to differential expression of its receptors. Previous studies show that the function of Unc5b and its other ligand, Netrin1, is highly variable depending on the dose and context (41–43). Netrin-1 acts through Unc5b to function as an endothelial survival factor, whereas unbound Unc5b increases endothelial cell apoptosis (44).

FLRT2 has only a short intracellular region but possesses an extracellular leucine-rich repeat motif that plays a central role in cellular behaviors, including protein-protein interactions, cellular adhesion or repulsion, migration, and growth (15, 45). In this respect, an important question remains: do molecular mechanisms related to the homophilic protein interactions of FLRT2 affect endothelial adhesion? Certain cytoskeletal proteins, or other cell adhesion molecules such as integrins, may be affected by homophilic FLRT2 binding, as indicated by a previous study (46). FLRT2 also interacts with growth factor receptors such as FGFR2 (47).

In summary, our findings show that FLRT2 provides noncanonical interendothelial adhesion through homophilic binding, which is exploited by abnormalized vessels within tumors. Our data also suggest that FLRT2 is possibly a novel theranostic molecule with potential utility as an effective and less harmful therapeutic target and/or as a biomarker for the long-term prognosis of patients with colorectal cancer.

## Methods

### Mice.

*Cdh-BAC-Cre^ERT2^* mice (48), which have been described previously, were mated with *Flrt2-flox* mice (16), *Vegfr2-flox* mice (49), *Unc5b-flox* mice (43), and *CAG-LSL-GFP* mice (50). Sex-matched *Cdh5-BAC-Cre^ERT2^Flrt2^fl/+^*, *Cdh5-BAC-Cre^ERT2^Vegfr2^fl/+^*, and *Cdh5-BAC-Cre^ERT2^Unc5b^fl/+^* mice were used as littermate controls. *APC^Min/+^* mice were obtained from The Jackson Laboratory (stock no. 002020). Mutant mice were crossed >10 times with C57BL/6J mice. Both sexes were used for the analysis of the phenotypes of mutant mice.

### Tumor models.

B16 mouse melanoma cells were cultured in DMEM containing 10% FBS. Cells (2 × 10^6^) were implanted subcutaneously into the backs of 4- to 6-week-old male mice. AXT mouse osteosarcoma cells were cultured in Iscove’s modified Dulbecco’s medium (Invitrogen) supplemented with 10% FBS and were implanted similarly. Tumors were measured daily with a caliper volume calculated using the following formula: *V* = tumor length × width × height/2. For Cre induction in *Cdh-BAC-Cre^ERT2^* mice, 100 μl 4OHT dissolved in oil at 5 mg/mL was injected into the inguinal fat according to the schedules shown in the figures. For some experiments, mice were injected intraperitoneally with 2.5 mg/kg per g bodyweight cisplatin dissolved in 100 μl DMSO or with the same volume of DMSO as a control, after tumors reached 30 mm^3^. For PD1 blockade, mice were injected intraperitoneally with 10 mg/kg per g bodyweight of an anti-PD1 mAb (BioXCell; BE0146) dissolved in 100 μl PBS; infections were given every 3 days, starting 6 days after tumor cell inoculation.

### Wound healing assay.

On 4 consecutive days, male mice (aged 4–6 weeks) were injected with 100 μl 4OHT dissolved in oil at 5 mg/mL (injections were administered into the inguinal fat). At 10 days after 4OHT injection, a full-thickness wound on the dorsal area was made in the shape of a circle measuring 10 mm in diameter. At 7 days after surgery, the wounds were resected (including a margin of normal surrounding skin) and fixed in 4% PFA. Paraffin sections were prepared as described previously and processed for Masson’s trichrome staining or fluorescent IHC.

### Preparation of whole-mount retinal samples.

Enucleated eyes were fixed for 20 minutes in 4% PFA in PBS and then dissected as described previously (48). Retinal cups were post fixed for 20 minutes and were stained as described below.

### Ischemic retinopathy model.

P8 mice with nursing mothers were maintained for 3 days in an atmosphere containing 85% oxygen and then placed back in room air as previously described (21). In some experiments, intraperitoneal administration of *N*-acetyl-L-cysteine (MilliporeSigma) dissolved in PBS (injection volume 0.5 g kg/body weight/d) was performed daily, from P11 to P15, in the ischemic retinopathy model.

### Generation of Flrt2^R186N+D188T^ mice.

Using the Optimized CRISPR design tool (Massachusetts Institute of Technology; http://crispr.mit.edu/), a CRISPR guide RNA (AGACTTGCAAGAGCTGAGAG) and an ssOligoDNA were designed to replace guanine 557 and alanine 558 in mouse *Flrt2* with alanine and cytosine respectively (c.557_558GA>AC), resulting in an amino acid substitution (p186Arg>Asn). In addition, guanine 562 and alanine 563 were replaced with alanine and cytosine (c.562_563GA>AC) to yield a second amino acid substitution (p188Asp>Thr). Next, gRNA-Cas9–expressing vectors (pX330-Flrt2-gRNA) were constructed. These gRNA-Cas9 vectors (30 ng/μl), along with ssOligoDNA (10 ng/μl), were comicroinjected into the pronuclei of fertilized mouse eggs. Surviving eggs were implanted into the oviducts of pseudopregnant foster mothers. Mice born to the foster mothers were genotyped by direct sequencing using the following primer: Flrt2FF-SEQ, 5′-TGGCAACCAGTTGGATGAGTTC-3′. PCR products were amplified using 2 primers: Flrt2FF-F, 5′-GCACACTGTCTACCTTTATGGCAAC-3′, and FLRT2FF-R, 5′-TGGTTATCCTGCAAGTAGAGCCTG-3′.

### Preparation of tissue sections and whole-mount samples.

Surgically dissected tissues were fixed overnight in 4% paraformaldehyde (PFA) in PBS. Frozen- or paraffin-embedded sections from those samples were used for immunohistochemical analysis. Before sectioning primary tumors derived from AXT cells, tumors were decalcified by overnight immersion in 0.5 M EDTA. All tumor samples were sectioned (14 μm thick for frozen samples or 3 μm for paraffin-embedded samples) at the plane of the maximum cut surface. To prepare whole-mount samples, tissues were dissected and fixed overnight in 4% PFA in PBS. All sections and whole-mount tissues were stained as described below.

### Immunostaining.

IHC of whole-mount samples or tissue sections was performed as described previously (51). The following primary monoclonal antibodies were used: hamster anti-CD31 (Chemicon, Temecula; MAB1398Z; 1:1,000), α-SMA-Cy3–conjugated (MilliporeSigma; C6198; 1:500), FLRT2 (R&D Systems; MAB2877; 1:500), VE-cadherin (Santa Cruz, sc-9989; 1:200), Ter119 (R&D Systems, MAB1125; 1:500), and Ki67 (Novus, NB600-1252; 1:500). The following primary polyclonal antibodies were used: anti-GFP–Alexa Fluor 488–conjugated (Molecular Probes,; A21311; 1:500), Collagen IV (Cosmo Bio, LSL-LB-1407; 1:500), and FLRT2 (R&D Systems, AF2877; 1:1,000). The secondary antibodies were Alexa Fluor 488–conjugated IgG (Molecular Probes, A11034, A11006, A11055; 1:500) and Cy3/Cy5 DyLight549/DyeLight649–conjugated IgG (Jackson ImmunoResearch; 711-165-152, 112-165-167, 127-165-160, 711-605-152, 112-605-167, 127-605-160, respectively; 1:500). Alexa Fluor 647–conjugated phalloidin (Invitrogen, A22287; 1:40) was used to visualize F-actin. To detect hypoxic cells, the Hypoxyprobe-1 Plus Kit (Chemicon, HP2-100) was used. Briefly, 60 mg/kg pimonidazole was injected intraperitoneally into mice 30 minutes before euthanasia. Sections were stained with Hypoxyprobe Mab1-FITC. For nuclear staining, specimens were treated with DAPI (Molecular Probes, D-1306). For angiography, 50 μl FITC-dextran (10 mg/mL; MW 2000 kDa; MilliporeSigma) in PBS was injected into the left cardiac ventricle and allowed to circulate for 3 minutes.

### Confocal microscopy.

Fluorescence images were obtained under a confocal laser scanning microscope (Olympus, FV1000). Quantification of cells or parameters of interest was conducted using 3 images, each with a 1270 × 1270 μm field of view, per sample (to count Ki67^+^ cells and vessels with tumor cell intravasation and to identify SMA^+^ vessels), or entire sections of tumor, lung, and liver (to count tumor vessels and to measure hemorrhagic, perfused, hypoxic, metastatic, and vascularized areas). ImageJ software (NIH) was used for quantification of the indicated areas.

### Sample preparation for ^13^C_6_-glucose metabolic pathway tracing analysis.

To trace the metabolic pathways of cancer cells, ^13^C_6_-glucose (1 mg/body weight [g]) was injected intraperitoneally into mice 30 minutes before resecting tumors (on the tenth day after implantation). Dissected tumors were frozen immediately and stored at –80°C until use. Tumor tissues were used for both metabolome analysis and IMS. Metabolite extraction for metabolome analysis was performed as described previously (52). Briefly, frozen tissue blocks and internal control compounds (2-morpholinoethanesulfonic acid) were homogenized in ice-cold methanol (500 μl) using a manual homogenizer (Finger Masher; AM79330), followed by addition of an equal volume of chloroform and 0.4 times the volume of ultrapure water (LC/MS grade, Wako). The suspension was then centrifuged at 15,000*g* for 15 minutes at 4°C. After centrifugation, the aqueous phase was filtered using an ultrafiltration tube (Human Metabolome Technologies; Ultrafree-MC, UFC3 LCC NB). The filtrate was concentrated in a vacuum concentrator (Thermo Fisher Scientific; SpeedVac), dissolved in 50 μl ultrapure water, and used for metabolome analysis.

### Metabolome analysis.

For metabolome analysis focused on glucose metabolic central pathways, namely glycolysis, the TCA cycle, and the PPP, anion metabolites were measured using an Orbitrap-type mass spectrometer (Thermo Fisher Scientific; Q-Exactive Focus) connected to a high-performance ion chromatography (IC) system (Thermo Fisher Scientific; ICS-5000+), which enabled performance of highly selective and sensitive metabolite quantification owing to the IC separation and Fourier Transfer MS principle (53). The IC device was equipped with an anion electrolytic suppressor (Thermo Fisher Scientific; Dionex AERS 500), which converts the potassium hydroxide gradient into pure water before the sample enters the mass spectrometer. Separation was performed using a Dionex IonPac AS11-HC, 4 μm particle size column (Thermo Fisher Scientific). The IC flow rate was 0.25 mL/min, supplemented post-column with a 0.18 mL/min makeup flow of methanol. The potassium hydroxide gradient conditions for IC separation were as follows: 1–100 mM (0–40 min), 100 mM (40–50 min), and 1 mM (50.1–60 min), at a column temperature of 30°C.

### Matrix coating and MALDI-IMS acquisition.

MALDI imaging analyses were performed as described previously (54). Briefly, thin sections (8 μm) of tumor were prepared with a cryomicrotome (Leica Microsystems; CM3050). Sections were attached onto indium tin oxide-coated glass slides (Bruker Daltonics GmbH) and coated with 9-aminoacridine as the matrix (10 mg/mL, dissolved in 80% ethanol) by manual spraying with an artistic brush (Procon Boy FWA Platinum, Mr. Hobby). The matrix was applied simultaneously to multiple sections to maintain consistent analyte extraction and cocrystallization conditions. MALDI imaging was performed using an Ultraflextreme MALDI-TOF/TOF mass spectrometer equipped with an Nd:YAG laser. Data were acquired in the negative reflection mode, with raster scanning at a pitch distance of 50 μm. Each spectrum was the result of 300 laser shots per data point. For TOF/TOF measurement, signals between *m/z* 50 and 1000 were collected. Image reconstruction for both procedures was performed using FlexImaging 4.1 software (Bruker Daltonics). Molecular identification was based on an accurate *m/z* value derived from FT-ICR-MS data and previous studies (54).

### ^13^C_6_-glucose metabolic pathway-tracing analysis.

2-DG (1 mg/body weight[g]) was injected intraperitoneally 30 minutes before resecting tumors (on the tenth day after implantation). The amount of 2DG-6P was quantified by IC-MS, and the tissue distribution was assessed by MALDI-IMS as described above.

### RT-PCR analysis.

Total RNA was prepared from cultured cells, or from CD31^+^ endothelial cells isolated from the B16 tumors using collagenase and dispase, and purified using Dynabeads (Veritas). Reverse transcription was performed using Superscript III (Invitrogen). cDNA samples were subjected to PCR amplification using the following primers: (a) mouse Flrt2, 5′-AGACAAGGCTGCCAGATTACA-3′ and 5′-TAAAATGCAACGTGATGGGG-3′; (b) mouse β-actin, 5′-ATGGATGACGATATCGCTGC-3′ and 5′-AGCACTGTGTTGGCATAGAG-3′. Quantitative PCR assays were performed on an ABI 7500 Fast Real-Time PCR System using TaqMan Fast Universal PCR master mix (Applied Biosystems) and TaqMan Gene Expression Assay Mix with human *FLRT2* (Hs00544171_s1) and *FLRT3* (Hs01922255_s1). A human *β-ACTIN* (Hs00357333_gl) assay mix served as an endogenous control. Data were analyzed using 7500 Fast System SDS Software 1.3.1.

### Cell culture.

HUVECs were cultured in EGM-2 medium (Takara-Bio) at 37°C/20% O_2_. In some experiments, 10 μM KU55933 (Calbiochem) dissolved in DMSO or 400 μM H_2_O_2_ was added to the culture medium, and cells were cultured for 24 hours.

### RNA interference.

For the RNA interference experiments, HUVECs were washed once with OptiMEM (Thermo Fisher Scientific) and transfected with 40 nmol/L siRNA Duplex (QIAGEN) using 6 μl/mL Lipofectamine RNAiMAX (Invitrogen) in OptiMEM. After 4 hours, the transfection medium was removed, and complete culture medium was added. Cells were cultured for a further 48 hours before use in in vitro assays. FlexiTube siRNA SI03061226 for FLRT2 (5′-CACGGUCUACCUGUAUGGCAA-3′; QIAGEN) was used to knockdown *FLRT2*. A human siRNA-negative control duplex oligonucleotide (5′-CUUACGCUGAGUACUUCGATT-3′, 5′-UCGAAGUACUCAGCGUAAGTT-3′; MilliporeSigma) was used as the negative control.

### RNA-Seq analysis.

The library for sequencing was prepared according to the manufacturer’s protocol (KAPA Biosystems; KAPA RNA HyperPrep Kit). Next, 2 × 150 bp pair-end sequencing was carried out using Illumina Hiseq. Sequencing data were converted into the fastq format using bcl2fastq software. The sequence quality was checked by FastQC software, and adapter sequences were eliminated by Trimmomatic. The sequence data were processed using the STAR-RSEM-DESeq2 pipeline. DEGs between HUVECs treated with si-Control and those treated with si-*FLRT2* were extracted by the Wald test using DESeq2 packages. TPM was used for the following analyses. The correlation matrix was calculated using Pearson correlation. Principle component analysis was performed using the scikit-learn package. A volcano plot was constructed using the bioinfokit package. GSEA was performed using the ontology gene sets in the Molecular Signatures Database (http://www.gsea-msigdb.org/gsea/msigdb/). IPA was performed to identify the most significant pathways for the DEGs (*q* < 0.01, logFC < –2 or > 5) in si-*FLRT2*–treated HUVECs compared with si-Control–treated cells. In brief, we uploaded the gene list file containing gene symbols and logFC; we then adjusted *P* values to the IPA and performed the core analysis. RNA-Seq data generated during this study are available at GEO (accession GSE179836).

### Adhesion assay.

Adhesion to ECM molecules was examined using a colorimetrically formatted CytoSelect 48-Well Cell Adhesion Assay Kit (Cell Biolabs). Briefly, cultured cells were suspended in serum-free medium at a concentration of 1 × 10^5^ cells/mL. Next, 150 μl of the cell suspension was added to each well and incubated for 60 minutes. Then, nonadherent cells were washed out, and 200 μl Cell Stain Solution (Cell Biolabs) was added to stain adherent cells. After washing away this solution, 200 μl Extraction Solution (Cell Biolabs) was added to each well, and the extracted sample was transferred to a 96-well microtiter plate to measure the optical density at 560 nm. Adhesion assays for the FLRT2 protein were performed using recombinant wild-type or mutant FLRT2 proteins. These proteins were diluted to the indicated concentration in PBS and coated onto 96-well flat-bottomed nontissue culture-treated polystyrene plates for 1 hour. Nonspecific sites were blocked for 1 hour at room temperature with 10 mg/mL BSA. After blocking, wells were washed with PBS. Next, 1 × 10^5^ HUVECs in 0.2 mL medium were added to each coated well. Plates were incubated at 37°C for 2 hours. Nonadherent cells were removed by washing with PBS, and the remaining cells were fixed for 30 minutes at room temperature in 4% PFA. Fixed cells were stained for 30 minutes with 0.5% crystal violet in 25% methanol and then solubilized in 1% SDS. OD_570_ values were measured.

### Human studies.

A cohort of 47 human malignant patients with colorectal cancer was analyzed to determine the expression pattern of FLRT2 in tumors. The 5-year RFS of another cohort (66 patients) with stage II/III cancer was analyzed alongside histological examinations. Sections (4 μm thick) of formalin-fixed and paraffin-embedded tissues were used for histological analysis. After deparaffinization and rehydration, sections were autoclaved at 121°C for 5 minutes in citrate buffer for antigen retrieval before immunostaining. To quantify FLRT2 expression, a 2200 × 1700 μm field of view in each section was examined under a light microscope, and the IHC score was determined objectively by 2 blinded researchers using the following formula: IHC score = 1 × (mildly positive vessels [%]) + 2 × (moderately positive vessels [%]) + 3 × (highly positive vessels [%]).

### Statistics.

All results are expressed as the mean ± SD. Comparisons between mean values of 2 groups were evaluated using a 2-tailed Student’s *t* test. Comparisons among multiple groups were evaluated using 2-way ANOVA followed by Bonferroni’s multiple-comparison test. Kaplan-Meier curves and the log-rank test were used to compare survival among the groups. Pearson’s correlation analysis was performed to determine correlations among groups. All calculations were performed using Stata 11.2 (Stata Corporation). *P* values of less than 0.05 were considered significant.

### Study approval.

Animal use and care were approved by the Institutional Animal Care and Use Committee of Keio University, and all experiments were performed in accordance with the Guidelines of Keio University for Animal and Recombinant DNA experiments. The human study was approved by the institutional ethics committee of Keio University Hospital (approval no. 20150051). A written informed consent was received from participants prior to their inclusion in the study.

## Author contributions

Y Kitagawa and Y Kubota designed the experiments. TA, Y Kitagawa, KO, YS, and ITN performed the experiments. TA, DK, KO, YS, ITN, Y Kido, and Y Kubota analyzed the data. HS, SN, DYL, ES, and SY provided experimental materials. KS, MS, ES, and SY edited the manuscript. TA and Y Kubota wrote the paper.

## Supplementary Material

Supplemental data

Supplemental table 1

## Version 1 02/01/2022

In-Press Preview

## Version 2 03/15/2022

Electronic publication
