# Supplementary material for: Tumor-specific interendothelial adhesion mediated by FLRT2 facilitates cancer aggressiveness
Source: J Clin Invest. 2022 Mar 15;132(6):e153626. doi: 10.1172/JCI153626 (PMC8920344; doi:10.1172/JCI153626)
Supplement: Supplemental data [file jci-132-153626-s065.pdf]

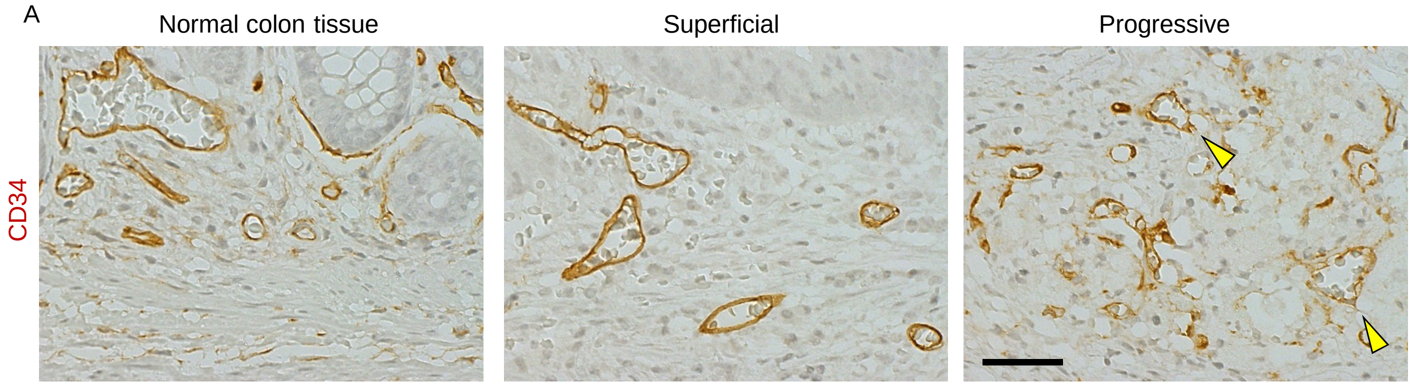

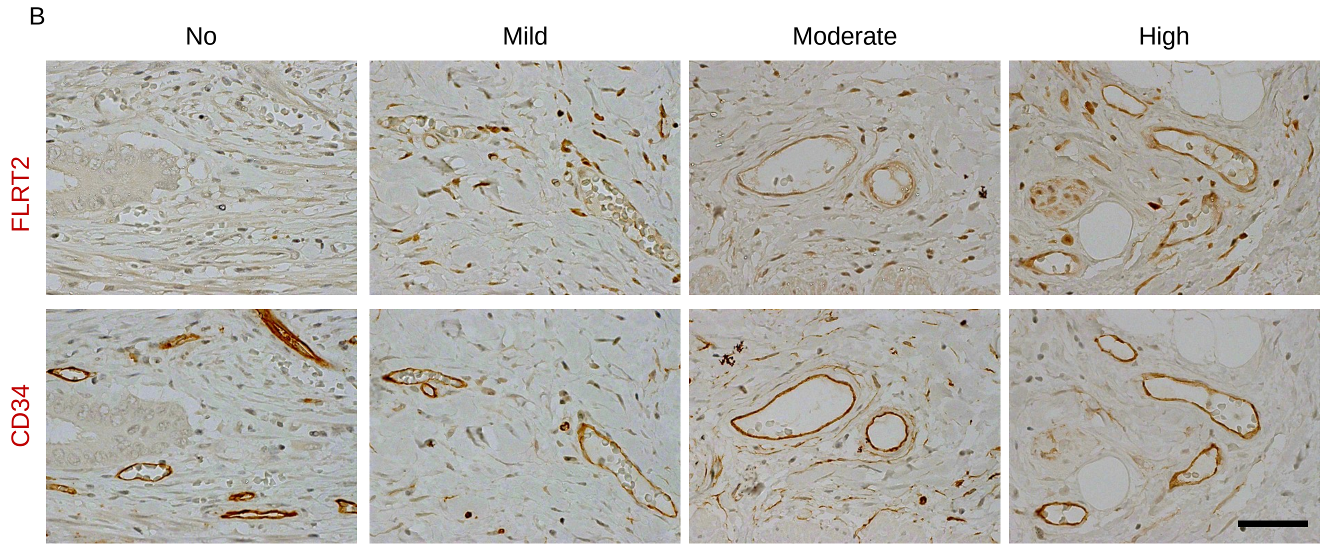

C

		n = 66
Age		69 (37 to 95)
Gender	Male / Female	36/30
PS	0-1 / 2-4	59/7
CEA		3.0 (0.3 to 156.1)
Tumor Primary Site	Colon / Rectum	50/16
Tumor Size (mm)		50 (10 to 120)
Stage	II / III	35/31
Tumor Depth	T1 or T2 / T3 or T4	2/64
Histology	tub or pap /<br>por or sig or muc	61<br>5
Vascular invasion		10/56
FLRT2 IHC score	IHC>3 / IHC<3	10/56

D

Cox proportional hazard model

	Univariate			Multivariate		
	HR	95%CI	*P*	HR	95%CI	*P*
Age	1.02	0.98, 1.07	0.28			
Gender (male)	0.73	0.24, 2.23	0.58			
Performance status (>1)	1.47	0.66, 3.26	0.35			
CEA	1.00	0.97, 1.03	0.87			
Stage (III)	6.98	1.54, 31.6	0.01	7.45	1.64, 33.8	0.01
Tumor primary site (Rectum)	0.55	0.12, 2.46	0.43			
Tumor size	0.98	0.96, 1.01	0.27			
Tumor depth (T3, 4)	0.24	0.03, 1.84	0.17			
Histology (por, sig,muc)	2.71	0.59, 12.4	0.20			
Vascular invasion (+)	2.28	0.30, 17.5	0.43			
FLRT2 IHC score (>3)	4.42	1.44, 13.6	<0.01	4.86	1.57, 15.0	0.01

Supplemental Figure 1

Supplemental Figure 1. Representative images for IHC score and characteristics of stage II/III colorectal cancer patients.

(**A**) Immunohistochemical analysis of CD34 expression in sections cut from resected tumors from stage IV cases. Arrowheads indicate sprouting-like structures. Representative images for three independent experiments are shown. (**B**) Representative immunohistochemistry images (n = 46) showing no (0), mild (1), moderate (2), and high expression of endothelial FLRT2. The immunohistochemistry score was determined objectively by two blinded researchers. (**C**) Characteristics of the 66 colorectal cancer patients examined in this study. (**D**) Univariate and multivariate analyses of stage II or III patients using Cox proportional hazards regression (n = 66). Scale bars: 50 µm. Comparisons between mean values of two groups were evaluated using a two-tailed Student's *t*-test.

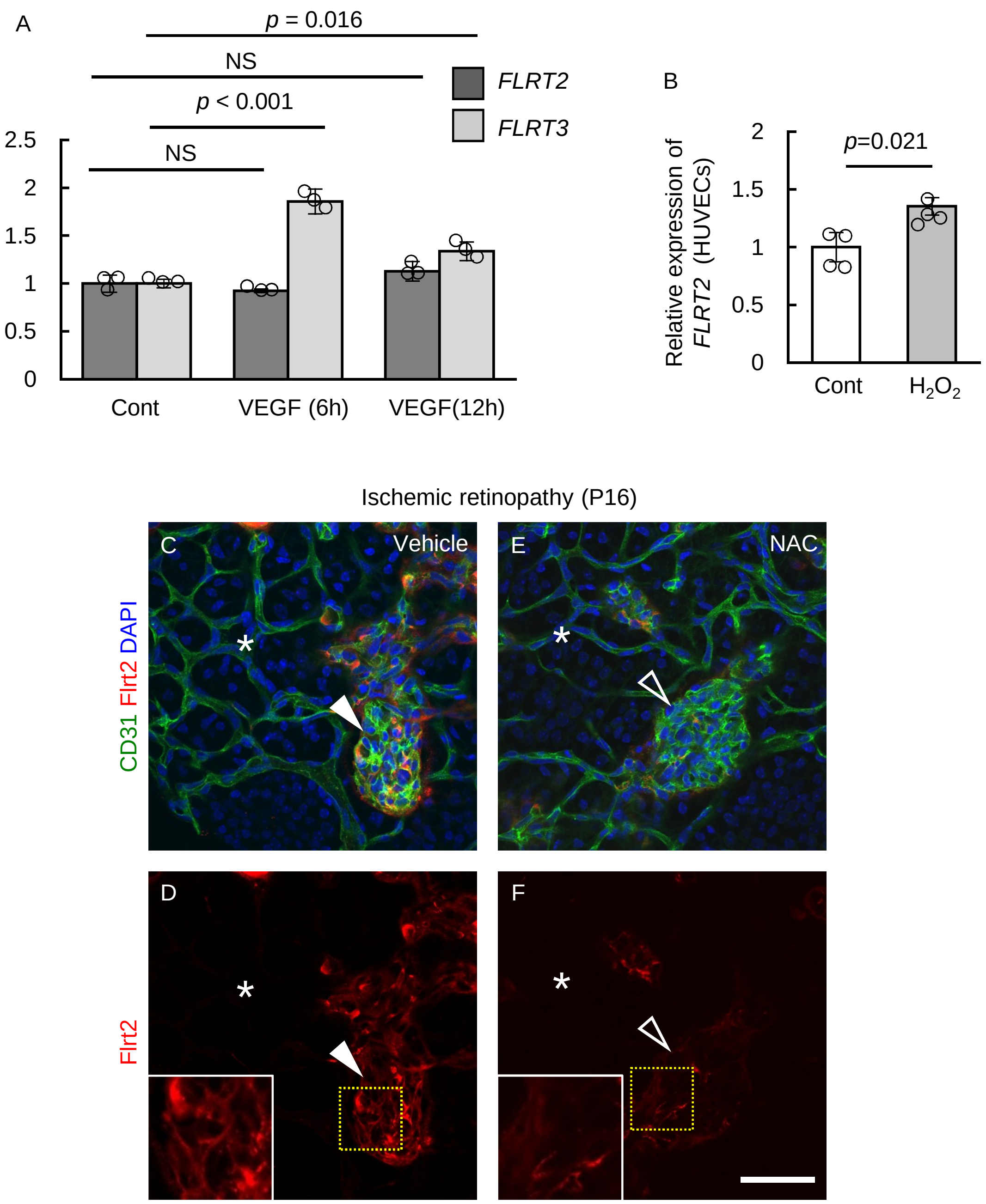

Supplemental Figure 2

Supplemental Figure 2. FLRT2 expression is induced by oxidative stress.

(**A–B**) Relative expression of FLRT2 in HUVECs under various culture conditions (n = 3, 4). (**C–F**) Whole-mount retina at P16 during ischemic retinopathy. Flrt2 is abundantly expressed in aberrantly expanding neovessels (crossed arrowheads), but not in surrounding stable vessels (asterisks). This expression of FLRT2 is suppressed by administration of the anti-oxidant, NAC. Representative images for three independent experiments are shown. Scale bar: 50 µm. Data are presented as the mean ± SD. NS, not significant. Comparisons between mean values of two groups were evaluated using a two-tailed Student's *t*-test. Comparisons between multiple groups (**A**) were evaluated using two-way ANOVA followed by Bonferroni's multiple comparison test.

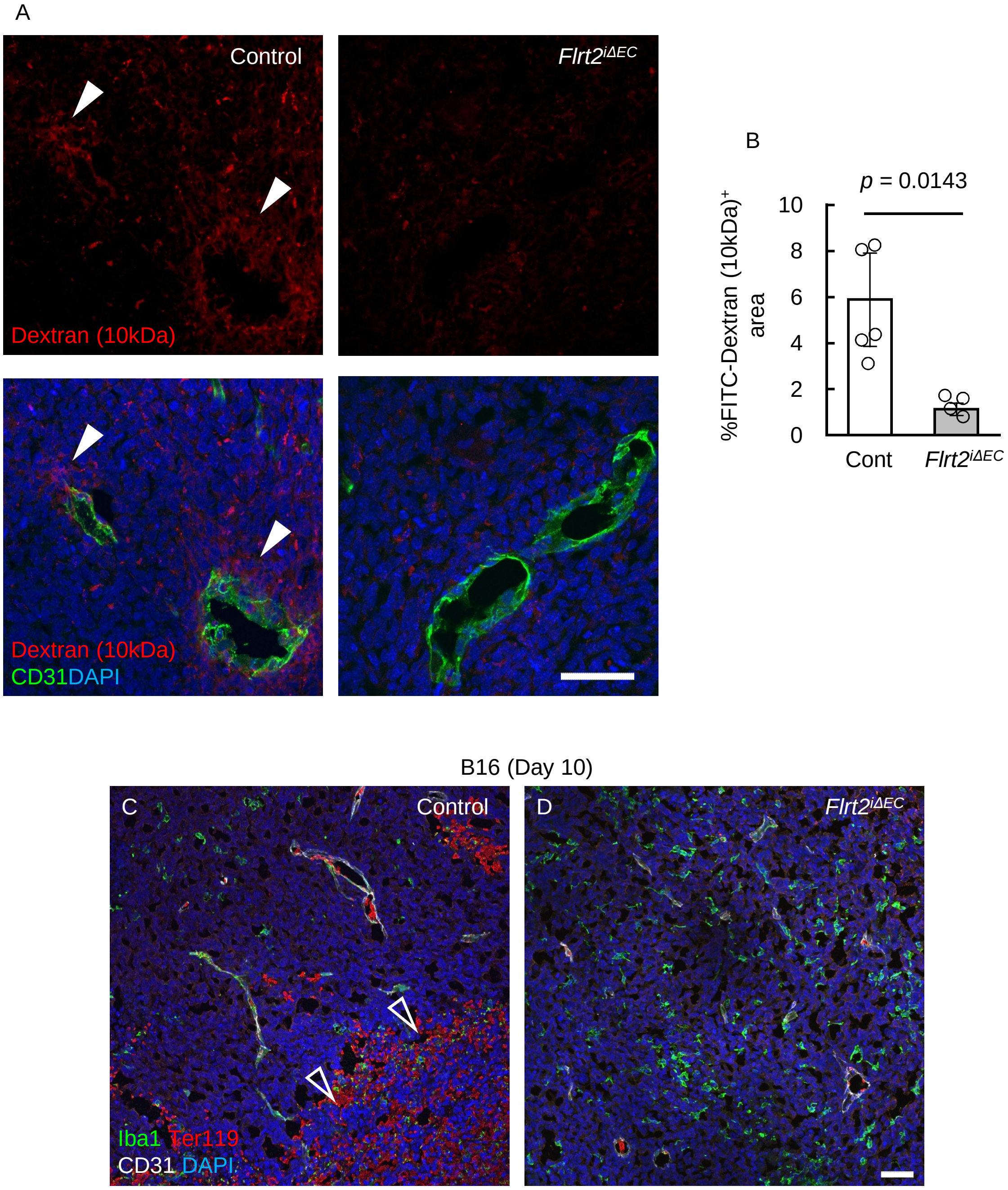

Supplemental Figure 3

Supplemental Figure 3. Vessel leakiness and macrophage accumulation affected by Flrt2 deletion.

(**A–D**) Immunohistochemical analysis of tumor sections and quantification (n = 5, 4). Leaked Dextran (10kDa) (closed arrowheads) in tumors from control mice is greater than that in *Flrt2*$^{i\Delta EC}$ mice. In the hemorrhagic lesion (open arrowheads) in control mice, accumulation of macrophages is not apparent. For **C, D**, Representative images for three independent experiments are shown. Scale bars: 50 µm. Data are presented as the mean ± SD. Comparisons between mean values of two groups were evaluated using a two-tailed Student's *t*-test.

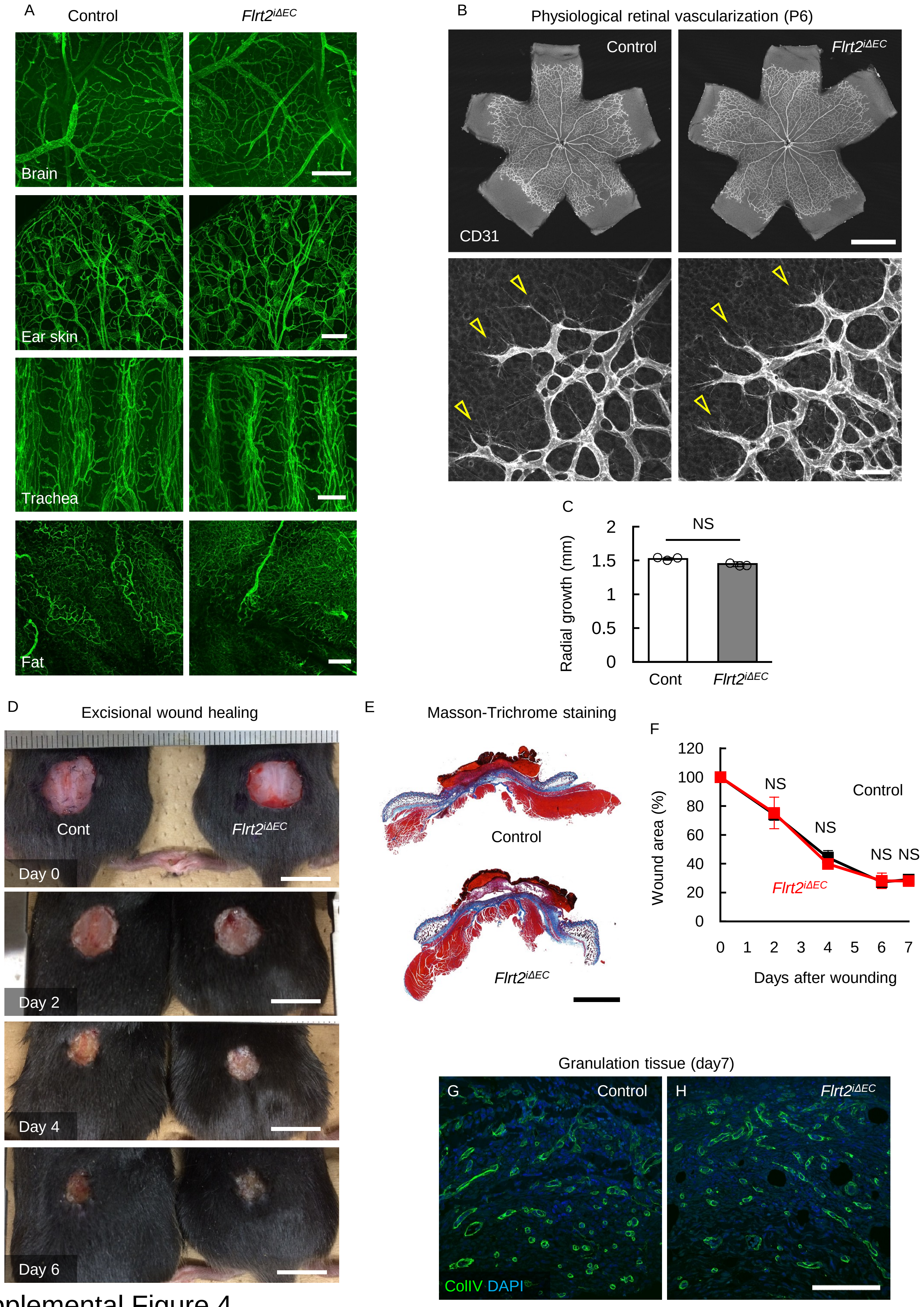

Supplemental Figure 4

Supplemental Figure 4. Normal vessels and wound healing are not affected by deletion of Flrt2.

(**A**) Whole-mount immunohistochemistry analysis of CD31 in normal organs from mice 10 days post-transplantation of B16 cells into the back skin. Representative images for three independent experiments are shown. (**B, C**) Whole-mount immunohistochemistry of P6 retina and quantification of radial growth (n = 3 each). The number of tip cells (arrowheads) was not affected. (**D–F**) Macroscopic appearance of the healing process after excisional wounding; Masson’s trichrome staining of wound sections was performed 7 days after wounding, followed by quantification (n = 3 each). (**G, H**) Immunohistochemical analysis of granulation tissues from mice 7 days after wounding. Representative images for three independent experiments are shown. Scale bar: 1 cm (**B (upper), D**); 2 mm (**E**); 200 µm (**A**); 50 µm (**B (lower), G, H**). Data are presented as the mean ± SD. NS, not significant. Comparisons between mean values of two groups were evaluated using a two-tailed Student’s *t*-test.

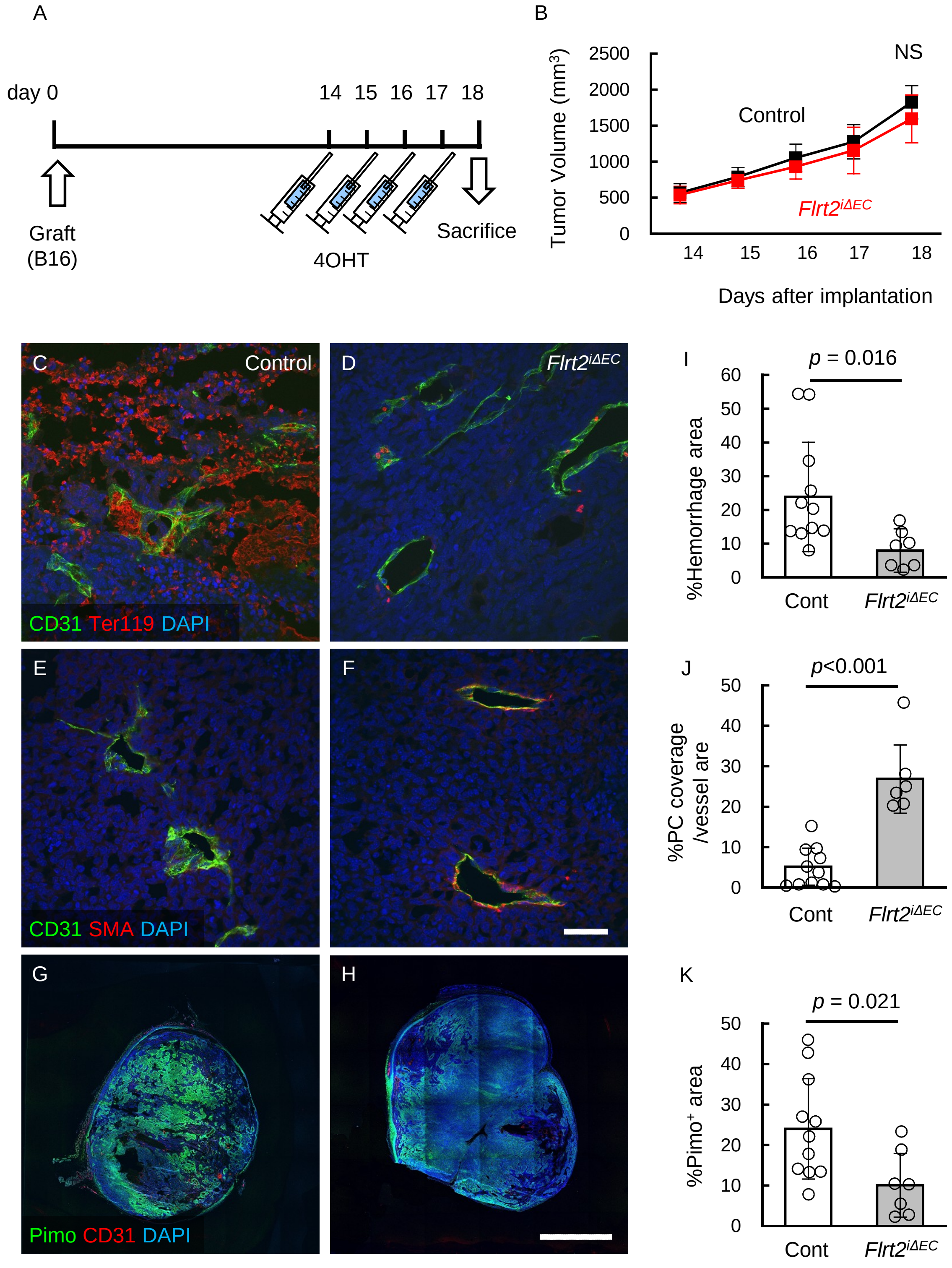

Supplemental Figure 5

Supplemental Figure 5. Intermittent Flrt2 deletion normalizes tumor vessels.

(**A**) Protocol for 4OHT injections and tumor inoculations. (**B**) Measurement of tumor volume (n = 11, 7). (**C–K**) Immunohistochemical analysis of tumor sections (n = 11, 7). Scale bars: 2 mm (**G, H**); 50 µm (**C–F**). Data are presented as the mean ± SD. NS, not significant. Comparisons between mean values of two groups were evaluated using a two-tailed Student's *t*-test.

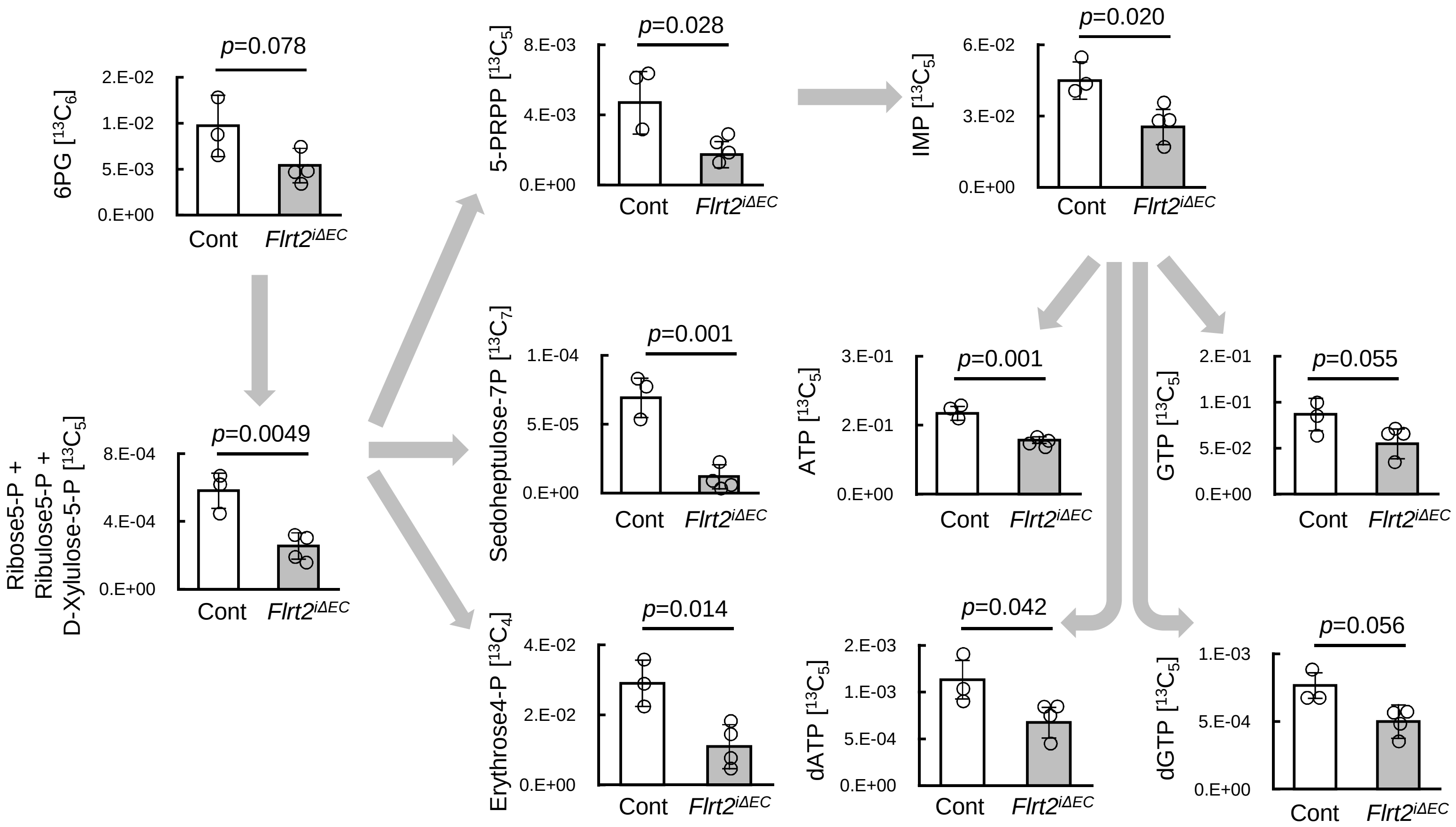

Supplemental Figure 6

Supplemental Figure 6. Deletion of endothelial Flrt2 inhibits the pentose-phosphate pathway in tumors.

Relative intracellular levels of the pentose phosphate pathway metabolites, as measured by IC-MS analysis (Peak area/IS/tissue-mg) (n = 3, 4). Data are presented as the mean ± SD. NS, not significant. Comparisons between mean values of two groups were evaluated using a two-tailed Student's *t*-test.

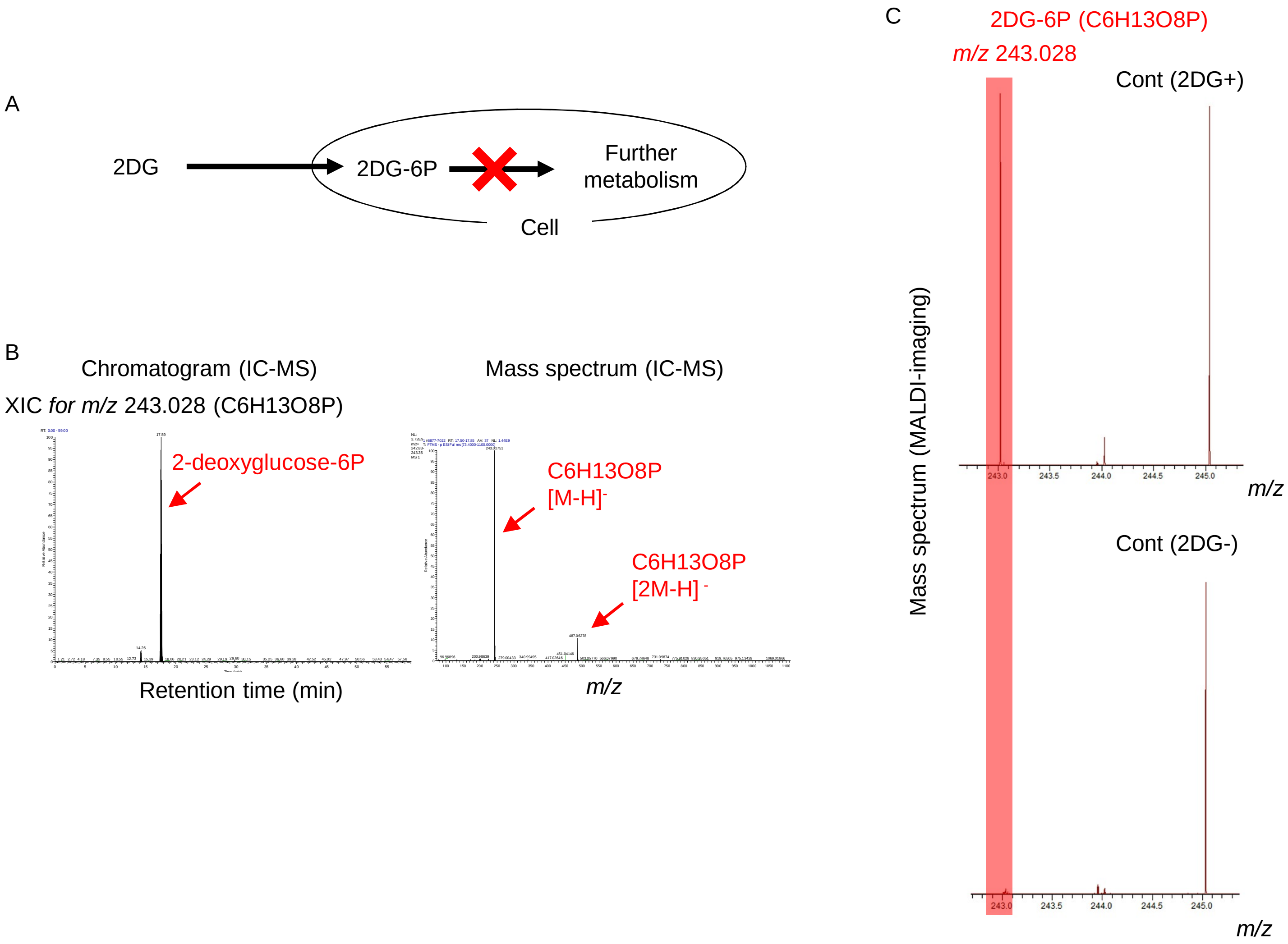

Supplemental Figure 7

Supplemental Figure 7. 2DG is converted intracellularly to 2DG-6P, which accumulates in the tumor.

(**A**) 2DG is taken up by cells and phosphorylated by hexokinase to yield 2DG-6P, which accumulates in the cells without further metabolism. (**B**) Ion chromatography analysis revealed that the chromatographic peak of 2DG-6P appeared predominantly at around 17 min; the mass spectrum obtained at this retention time confirmed that the signal was derived from 2DG-6P. Representative chromatograms for three independent experiments are shown. (**C**) The tumor tissue used for IC-MS was cut out and analyzed directly by MALDI-MS using an Orbitrap-type mass spectrometer; a signal corresponding to 2DG-6P was observed at m/z 243.028 only when 2DG was administered. Representative chromatograms for three independent experiments are shown.

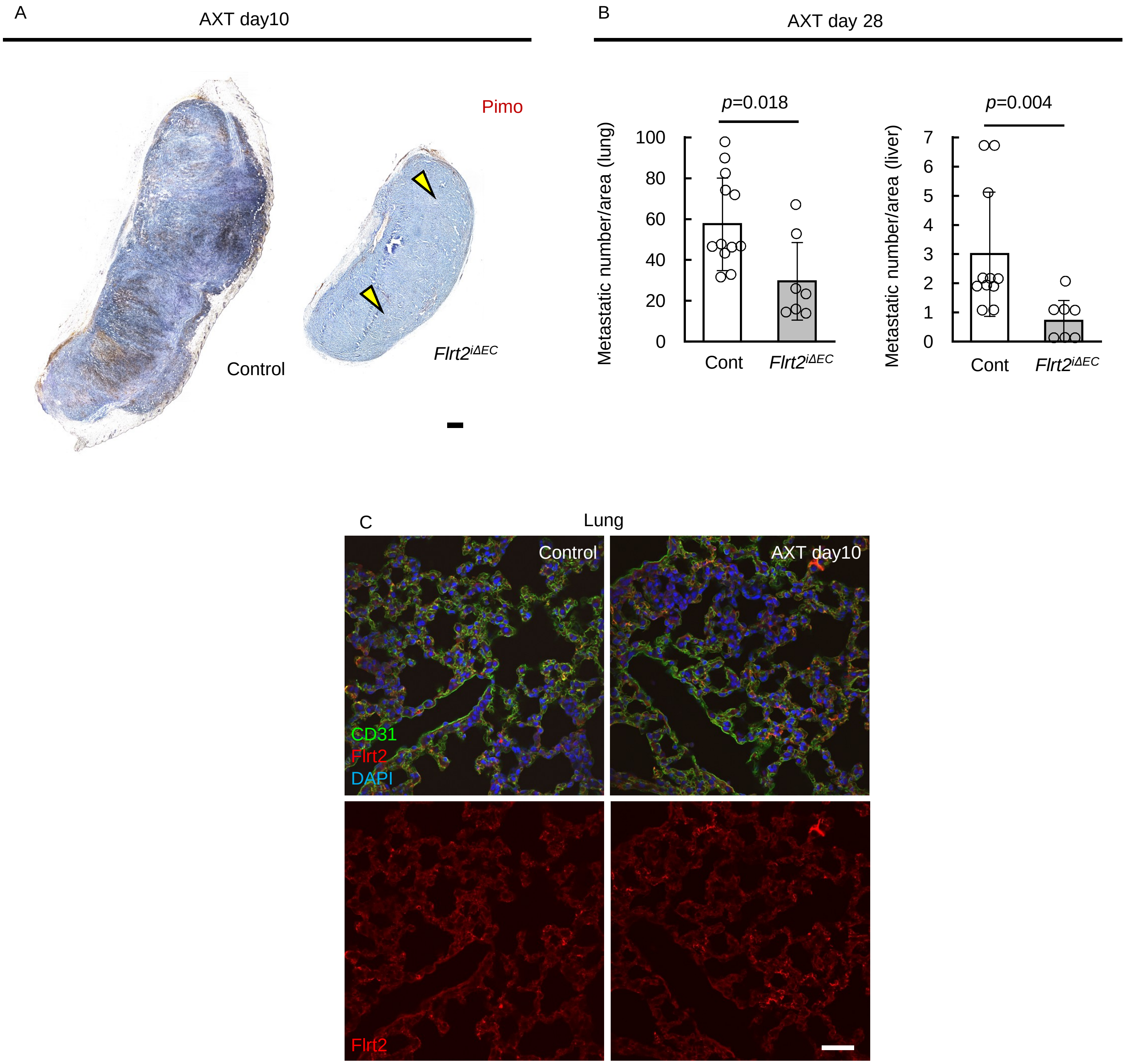

Supplemental Figure 8

Supplemental Figure 8. Tumor Metastasis and hypoxia affected by Flrt2 deletion.

(**A**) Immunohistochemical analysis of AXT tumors. Tumors from *Flrt2*$^{i\Delta EC}$ mice show reduced hypoxia (closed arrowheads). Representative images for four independent experiments are shown. (**B**) Quantification of the number of metastatic colonies 28 days after transplantation of AXT cells into the back skin (n = 12, 7, 11, 7). (**C**) Immunohistochemical analysis of lungs before and after (10 days) post-transplantation of AXT cells into the back. Representative images for three independent experiments are shown. Scale bar: 500 µm (**A**); 50 µm (**C**). Data are presented as the mean ± SD. NS, not significant. Comparisons between mean values of two groups were evaluated using a two-tailed Student's *t*-test.

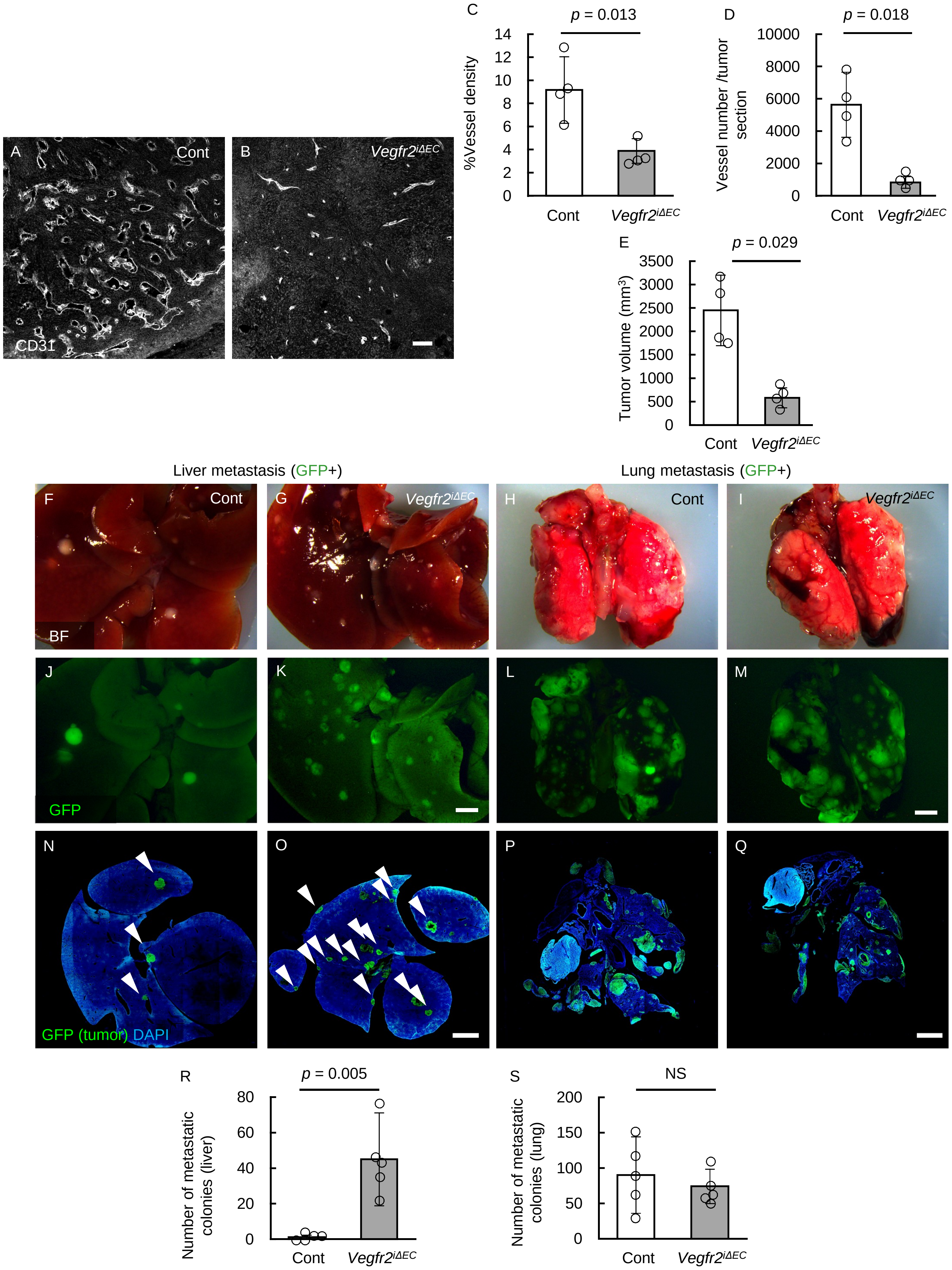

Supplemental Figure 9

Supplemental Figure 9. Deletion of endothelial Vegfr2 increases tumor metastasis.

(**A–E**) Immunohistochemical analysis and quantification of primary tumors in mice 28 days after transplantation of AXT cells into the back skin (n = 4 each). Tumor vessels in *Vegfr2*$^{i\Delta EC}$ mice are tortuous and fragmented. (**F–S**) Macroscopic appearance and immunohistochemical analysis of livers and lungs from mice 28 days after transplantation of AXT cells into the back skin (n = 5 each). The number of metastatic colonies (arrowheads) in the liver of *Vegfr2*$^{i\Delta EC}$ mice is higher than that in littermate controls. Scale bars: 2 mm (**F–Q**); 50 µm (**A, B**). Data are presented as the mean ± SD. Comparisons between mean values of two groups were evaluated using a two-tailed Student’s *t*-test.

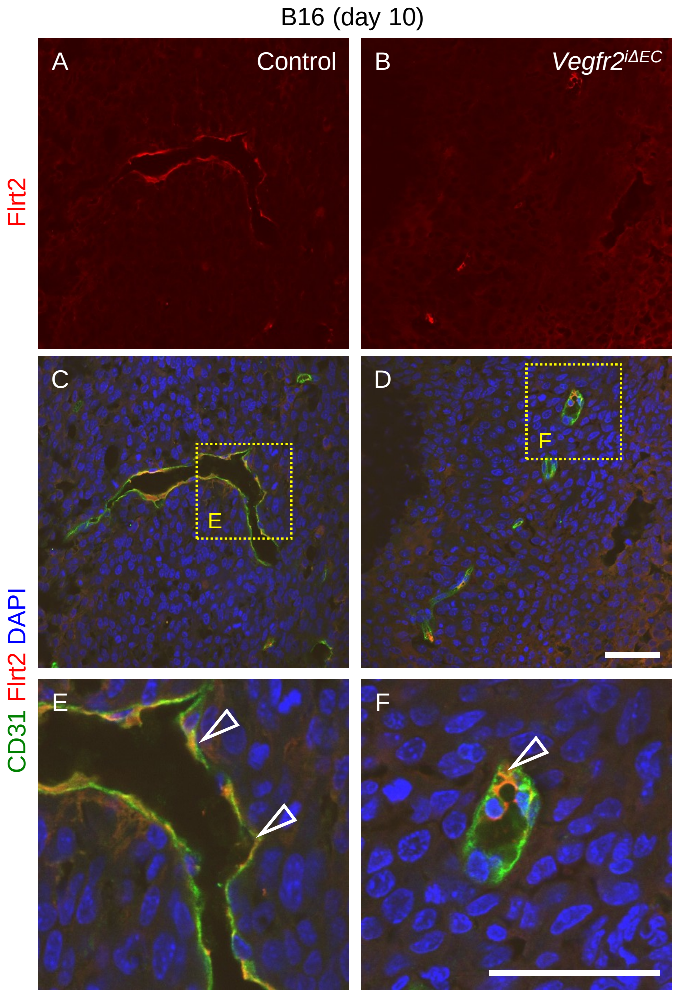

Supplemental Figure 10

Supplemental Figure 10. Deletion of endothelial Vegfr2 does not affect the expression of Flrt2.

(**A–F**) Immunohistochemical analysis of tumors in mice 10 days after transplantation of B16 cells. The expression of Flrt2 in the inter-endothelial junctions (arrowheads) is intact in tumors of *Vegfr2*$^{i\Delta EC}$ mice. Representative images for three independent experiments are shown. Scale bars: 50 µm.

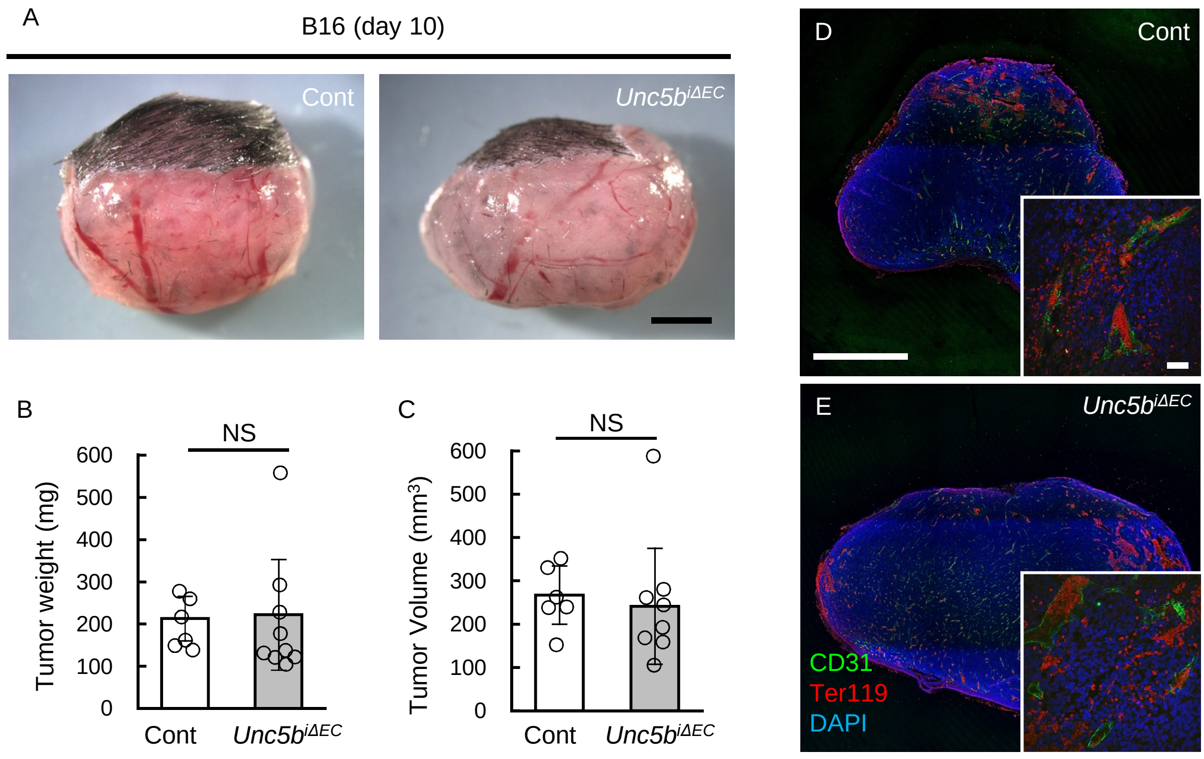

Supplemental Figure 11

Supplemental Figure 11. Deletion of endothelial Unc5b does not affect tumor angiogenesis in the B16 melanoma model.

(**A–C**) Bright field views and quantification in tumors 10 days after inoculation of B16 melanoma cells. 4OHT was injected for 4 consecutive days after tumor inoculation. (**D, E**) Tumor sections stained with the indicated antibodies. Neither vessel number nor hemorrhage was affected by deletion of endothelial *Unc5b*. Representative images for five independent experiments are shown. Data are presented as the mean ± SD. NS, not significant. Scale bar: 2 mm (**A, D, E**); 200 µm (insets in **D, E**). Comparisons between mean values of two groups were evaluated using a two-tailed Student's *t*-test.

A Genes upregulated in si-*FLRT2*

	Base Mean	log_2FC	*p*-value
NEURL3	540.45	11.87	3.74E-31
RUNX3	256.86	10.62	7.19E-25
CXCL10	5804.27	10.23	0
NKD2	117.76	10.18	1.38E-22
LINC02605	100.86	10.17	9.23E-23
CSF2	143.44	9.96	6.46E-22
TNFAIP6	81.25	9.49	8.57E-20
KLHDC7B	103.18	9.48	9.77E-12
MSC	157.31	9.27	2.54E-23
DCLK3	51.83	9.07	2.68E-17
GATA5	69.00	8.90	1.10E-17
RSAD2	12510.27	8.76	0
CLIC6	36.36	8.71	3.16E-16
AC083837.1	55.95	8.60	1.88E-16
BIRC3	7476.19	8.49	7.65E-222
SLC22A1	21.83	8.45	1.19E-12
FOXF2	27.25	8.29	1.27E-14
IDO1	304.17	8.28	1.51E-56
RPLP0P2	160.05	8.26	2.13E-41
IRX2	24.93	8.16	4.89E-14
MC5R	26.00	8.11	1.77E-13
ATP10A	53.53	8.09	4.39E-15
RET	286.16	8.09	2.24E-60
NOD2	48.20	8.06	1.50E-15
MSC-AS1	27.13	7.96	2.20E-13
RTP4	144.81	7.94	3.20E-33
CXCL11	2949.84	7.93	0
OR2I1P	21.44	7.92	1.75E-12
GBP1P1	18.67	7.88	7.38E-13
CCL5	929.50	7.75	7.66E-213
TNFRSF9	324.83	7.66	3.14E-93
PID1	35.66	7.64	5.16E-13
DCDC2C	13.28	7.59	2.96E-10

B

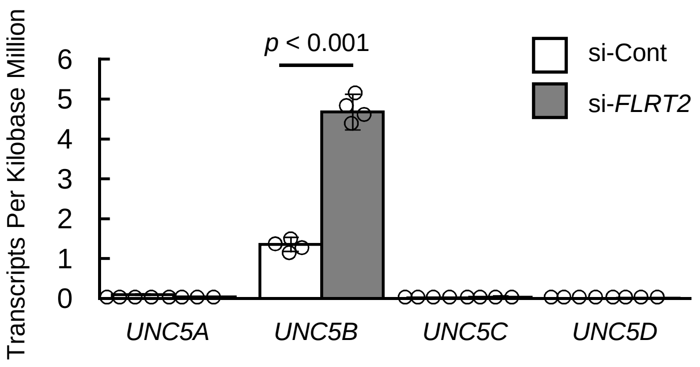

Supplemental Figure 12

Supplemental Figure 12. Differentially expressed genes and expressions of UNC5 receptors in si-FLRT2-treated HUVECs.

(**A**) Top 33 genes upregulated by si-*FLRT2*. Genes highlighted in green are related to regulation of reactive oxygen species or apoptotic signaling pathways. (**B**) The absolute mRNA amounts of these genes (Transcripts Per Kilobase Million) in HUVECs. The expression levels of *UNC5A, B, C,* and *D* were extracted from the raw RNA-seq data (n = 4). Data are presented as the mean ± SD. Comparisons between mean values of two groups were evaluated using a two-tailed Student's *t*-test.

Supplemental Table

Supplemental Table 1. List of differentially expressed genes identified by RNA sequencing.
